# Buffering effects of supporting electrolytes on pH profiles in electrochemical cells

**DOI:** 10.1038/s41598-025-18219-z

**Published:** 2025-09-12

**Authors:** Benjamin Janotta, Maximilian Schalenbach, Marcel Turiaux, Hermann Tempel, Rüdiger A. Eichel

**Affiliations:** 1 Fundamental Electrochemistry (IET-1), Forschungszentrum Jülich, Institute of Energy Technologies, Wilhelm-Johnen-Straße, 52425 Jülich, Germany; 2 Institute of Physical Chemistry, RWTH Aachen University, 52062 Aachen, Germany; 3 Faculty of Mechanical Engineering, RWTH Aachen University, Aachen, Germany

**Keywords:** Local pH, pH indicator, Buffers, Electrolytes, Ion transport, Transport properties, Simulation, Chemical engineering, Electrochemistry, Theoretical chemistry

## Abstract

In electrochemical processes, the pH values at the electrodes affect their potentials, reaction kinetics, product compositions, etc. However, predicting local pH values is complicated due to multi-ion movement between the electrodes and buffering effects of the electrolyte constituents. Here, local pH values in an electrochemical cell are studied with a combined model and experimental approach, focussing on buffering effects of supporting electrolytes. The simulated ion displacements are evaluated with optically measured pH values using a thymol blue pH indicator in a Na_2_SO_4_-based electrolyte. By including buffering reactions of the pH indicator in the ion transport simulation, the local pH value in the near-neutral area and its temporal change are in precise agreement with the measurements. Without this buffering effect, the simulated propagation velocities of the pH fronts increase as such homogeneous reactions are shown to reduce the ion fluxes and those of the accompanied reaction products. The effect of the ionic strength on the ion transport in multi-ion electrolytes is included by modelling electrolyte transport properties with the Mean Spherical Approximation. Finally, the effects of current density and supporting electrolyte concentration on local pH values are elucidated based on a parameter variation of the simulations presented.

**Supplementary Information:**

The online version contains supplementary material available at 10.1038/s41598-025-18219-z.

## Introduction

In electrochemical applications with aqueous electrolytes like electroplating^1^, electrowinning^2^, batteries^3^, the chloralkali process^4^, aqueous CO_2_-electrolysis^5,8^, and water electrolysis^9^, the pH close to the electrodes significantly affects reaction kinetics and overpotentials. The pH at the electrodes directly affects process efficiencies^10^ and can be adjusted by buffer solutions^11^. However, the pH is a local and time-dependent property that is shaped by the electrolyte composition, buffering capacity of the electrolyte, electrode reactions, and ion transport^12^. Moreover, the process conditions in terms of temperature, current density, cell geometry, etc. affect pH gradients in electrolytes^13^. Hence, this large parameter space and its interdependences complicate an experimental-based optimization of electrochemical cells with pH gradients. Alternatively, accurate electrochemical transport simulations display powerful tools to understand these interdependencies and to optimize electrochemical processes with pH gradients.

Developing accurate models to predict local pH values is challenging due to three main reasons: (i) The ion transport properties required to simulate the ion movement are accessible for many binary electrolytes^14^ whereas such data for multi-ion electrolytes (denoting more than 2 ions in solution) is scarce^15^. Deconvoluting the contributions of the individual ions to the overall ion transport based on the combined macroscopic electrolyte properties is not straightforward as such estimations rely on fitting parameters that are specific to the considered ion combinations^16,17^. (ii) Electroneutrality must be maintained in the electrolyte during electrochemical transport simulations. However, the computation of electroneutrality and ion transport with multi-ion electrolytes in two or three dimensions comes with an algebraic constraint^18^. To overcome this technical hurdle, the electroneutrality constraint is often simplified or neglected, leading to unphysical simulation outcome^19^. (iii) To validate and improve the predictive capabilities of simulations, precise in situ measurements of the local pH values are required.

Precise measurements of local pHs remain an active area of research, conducted by electrochemical techniques^19^, optical methods^20^, and vibrational spectroscopy^21^. Electrochemical techniques, such as scanning probe methods and rotating ring-disc electrode voltammetry offer fast and easy insights but affect the local pH^22^. Yin et al.^23,24^ developed simple analytical expressions for steady-state applications relating the difference between bulk and surface pH with the current density. Critelli et al.^22^. have highlighted the potential artefacts caused by probe effects during pH measurements, particularly in transient processes, showing the demand for non-invasive methods. Since the 1970s, optical measurement techniques have been extensively developed for in situ and ‘minimally-invasive’ local pH measurements^25,26^. As outlined in the comprehensive review by Steinegger et al.^25^, optical techniques (being classified there into eleven distinct principles including for example infrared and spectroscopic sensing, absorptiometry and reflectometry, and luminescence-based methods) have to be adapted to individual experimental requirements. For example, identifying suitable pH-sensitive dyes that remain stable under process conditions, including varying pH ranges and exposure to possible electrochemical oxidation, poses a significant challenge. If pH sensitive dyes (including pH indicators) are used, these can intrinsically interact with the pH value^27^.

In recent studies^25–27^, simulated and measured pH values show large differences. These inconsistencies reflect the challenges from both sides, simulations and experiments. For instance, Pande et al.^27^. used confocal fluorescence microscopy with fluorescein additives to optically measure pH values between 5 and 9. With this approach, simulated and measured pH gradients deviated even at current densities in the range of $\:{\upmu\:}\text{A}/\text{c}{\text{m}}^{2}$. Yang et al.^28^. measured significant pH shifts (> 5 pH units) from bulk to electrode surface during buffered CO_2_ electrolysis at considerably low current densities (with respect to industrial applications) but could not match their model with the experiments due to the complexity of the investigated system. For example, gas bubble formation significantly impacts local pH values^29^. All studies found that compare modelled and measured pH gradients used a constant parametrization (often based on data at infinite dilution) for the transport properties^22,27,30–36^, which is known to reduce the simulation precision^18,37^. Thus, with the current state of the literature, a precise simulation of pH gradients is not possible.

Here, local pH values in buffered electrolytes are investigated with a combined model and experimental approach. To overcome the above listed drawbacks of previous studies, the transport properties (ionic conductivities and diffusion coefficients) are modelled concentration-dependent based on the Mean Spherical Approximation^38^. Moreover, a new design for the electrochemical cell is used that enables optical pH measurements at high pH gradients. Convection by instable, ion transport-driven density gradients is mostly suppressed by a vertical cell design. Bubbles are avoided by the design of the electrode reactions. Hence, the pH in a 1 M Na_2_SO_4_ supporting electrolyte could be precisely measured, serving as accurate reference for the ion transport model. With these approaches, the simulated time-evolution of the ion displacement precisely agrees with the experimental data. Intrinsic homogeneous reactions of pH-dyes are shown to significantly impact the ion transport, even if used in considerably low concentrations. For more complex industrial electrochemical cells, convective electrolyte and bubble transport remain a topic to be addressed in future studies.

## Results and discussion

### Comparison of optically evaluated experiment and simulations

As discussed in the “Methods” section in detail, the local pH is optically measured in an electrochemical cell with glass windows using thymol blue as pH indicator, which shows a sensitive colour transition from yellow to blue between pH 8 and 9.6. At the cathode of the reactor, oxygen is reduced in the gas diffusion electrode (GDE), which leads to hydroxide ion formation and/or proton consumption with respect to the local pH. Consequently, the cathode environment locally becomes more alkaline. At the anode, hydrogen is oxidised (proton formation and/or hydroxide ion consumption with respect to the local pH), locally acidifying the electrolyte. In the examined electrochemical cell, the range between pH 8 and 9.6 is typically spatially restricted to a sub-millimetre zone. This “transition zone” is in focus of the following optical evaluation of the electrolyte. In the following, the location of the transition zone is quantified by its distance *d* to the cathode. As thoroughly elucidated in the “Methods” section, the modelled ion transport is calculated based on the Nernst-Planck equation. The electrolyte transport properties (conductivities, diffusion coefficients, etc.) are either described by the values at infinite dilution, or the Mean Spherical Approximation (MSA). The homogeneous equilibria are calculated after the mass transport and assumed to equilibrate faster than the time increment of the simulation (in every time step the equilibrium of the homogeneous reactions is restored in each computational cell).

Four models for the electrolyte properties are compared within this study, as summarised by the overview in Table 1 that specifies the characteristics of each model. The simulations differ regarding the modelled species (where “Thb” denotes the dianionic form of thymol blue), how homogeneous reactions are considered, and how the ion properties are calculated. Water dissociation is always considered with a pK_a_ of 14. Like for H_2_SO_4_, homogeneous reactions of thymol blue (pK_a,1_ = 1.7, pK_a,2_ = 8.9) and H_2_CO_3_ (pK_a,1_ = 6.52, pK_a,2_ = 10.40) are considered.

**Table 1:** Model properties for the transport simulations including the respective considered species and interaction models. “Thb” denotes the dianionic form of thymol blue.

Simulation name	Modelled species	Homogeneous reactions considered?	Property model
S1	Na^+^, SO_4_^2−^, H^+^, OH^−^	No	Infinite dilution
S2	Na^+^, SO_4_^2−^, H^+^, OH^−^, HSO_4_^−^, H_2_SO_4_	Yes	Infinite dilution
S3	Na^+^, SO_4_^2−^, H^+^, OH^−^, HSO_4_^−^, H_2_SO_4_	Yes	MSA
S4	Na^+^, SO_4_^2−^, H^+^, OH^−^, HSO_4_^−^, H_2_SO_4_, Thb^2−^, HThb^−^, H_2_Thb	Yes	MSA

**Fig. 1:**
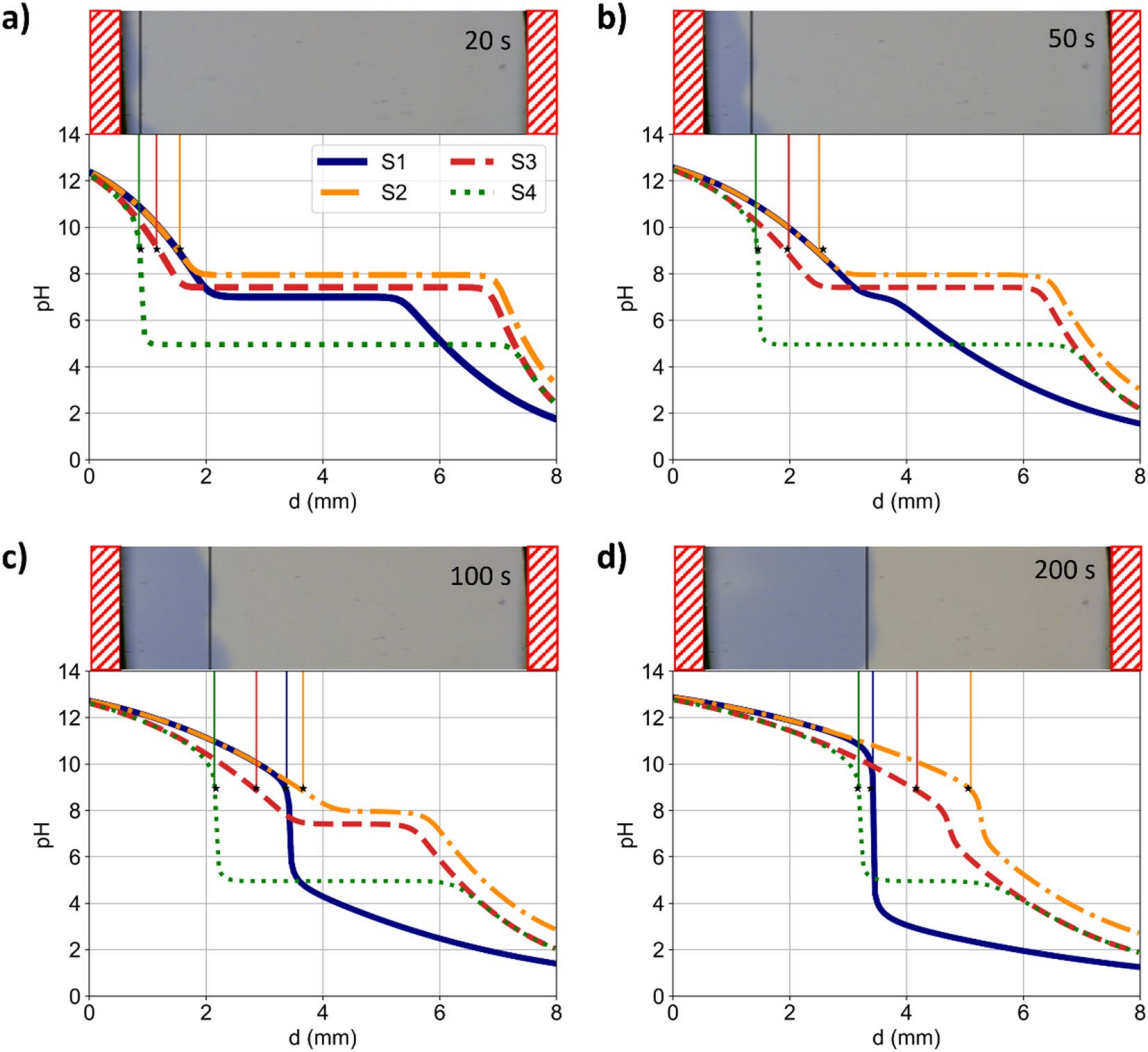
Comparison between experimental data (top, photo) and simulations (bottom, graph) using the parametrisations S1 to S4 (see Table 1) after (**a**) 20 s, (**b**) 50 s, (**c**) 100 s, (**d**) 200 s. For each of the different times, a photo of the electrolyte is shown on top, while the modelled pH profiles are graphed below. The photos (see original data in the Supporting Information) are squeezed horizontally and turned by 90 degrees. Hence, the distance d shows the distance from the cathode. The red shaded areas indicate the non-observable areas covered by sealings. The vertical lines in the plot of the simulation results enable an optical comparison with the experimental evaluation.

Figure 1 shows four exemplary comparisons of the modelled and measured pH values after 20, 50, 100, and 200 s after the current density of 3.33 mA/cm^2^ was switched on. For each of these times, pictures of the electrolyte (rotated by 90 degrees) from the experiments and the simulated ion displacements are shown. The used model parameterizations for all simulations (S1 to S4) are summarised in Table 1. The initial pH values of S1 to S4 are 7.0, 8.0, 7.4, 5.0. For all these pH values, thymol blue shows a yellowish discoloration. In Fig. 1a the propagation of the alkaline phase from the top (left) is shown by the blue discoloration. S1 and S2 (transport properties at infinite dilution) show the fastest simulated propagation of the transition zone, deviating significantly from the experimental data. The transition zone of S1 stays at $\:{d}_{\text{S}1}=3.2$ mm as visible in Fig. 1c and d, while the transition zone of S2 propagates further towards the anode. S3 shows a slower propagation of the transition zone due to decreased mobilities resulting from ion-ion interaction considered *via* the MSA. Lastly, S4 shows the slowest propagation due to the homogeneous reaction of thymol blue that are additionally considered.

Like the pH indicator, dissolved CO_2_ from the air slows down the propagation of the pH gradient (see Supplementary Information). In the experimental data, the transition zones are indicated, showing slight variations of the penetration depths. However, the uncertainty of the measured location of the transition zone is approximately $\:\pm\:$0.25 mm, which displays only a fraction of the evaluable optical window. The effect of this uncertainty is considered further in Fig. 2.5 in the Supporting Information. The variation of the location of the transition zone along the reactor width in the experiment may be explained by inhomogeneous reaction rates at the gas-diffusion electrodes (GDE). Moreover, natural convection resulting from density gradients may disturb the propagation front. Instable density layers may occur due to the displacement of ions with different ion species having different excess volumes. Furthermore, the heat of formation of water from H^+^ and OH^−^ inside the electrolyte facilitates natural convection. In preliminary experiments, the anode was placed on top, while the cathode was placed at the bottom. Thus, high density electrolyte layers on top and low-density electrolyte area at the bottom were formed, leading to vivid natural convection. With the vertical stacking employed for the presented measurements, convection-related effects were minimised, allowing the experimental results to closely match the one-dimensional MSA-based simulation S4. This close match demonstrates that the related challenges for matching simulations and experiments reported in the literature^27,28^ were effectively addressed.

**Fig. 2:**
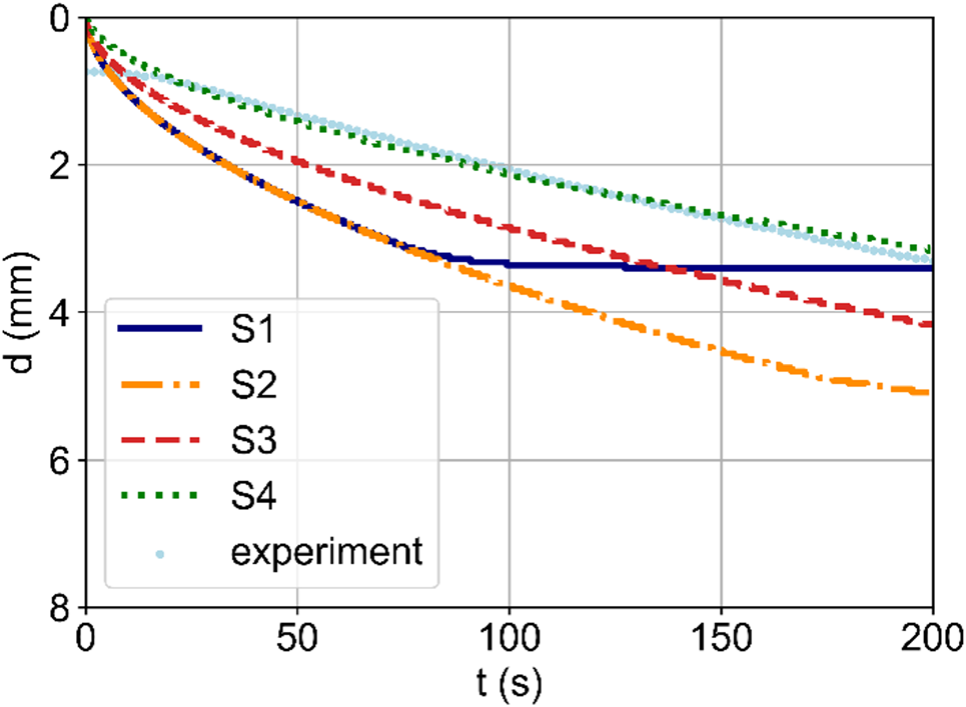
Time-evolution of the distance d of the transition zone (pH 8 to pH 9.6) to the cathode as evaluated from the experiment (scatter) and simulations S1 to S4 (lines).

Figure 2 shows the time-evolution of the location of the transition zone $\:d$ for the experiment and the four simulations conducted. The evaluated experimental data do not start at 0 mm, which is due to the non-observable area. The simulation S1 (transport properties at infinite dilution and neglected buffer reactions) predicts a stationary distance for the transition zone at $\:d=3.4$ mm after 90 s. By further including the equilibrium constants of the sulphate ion based on concentrations (simulation S2), the propagation of the transition zone initially follows the same trend as S1, but the alkaline phase propagates further towards the anode as the effective mobility of the protons (coming from the anode) is reduced due to the equilibrium reactions. With simulation S3, the MSA-model is added. As a result, the transition zone propagates slower towards the anode than using S1 and S2. By additionally including the buffer reactions of thymol blue (simulation S4), the slowest propagation of the alkaline phase and the best match with the experimental data is obtained.

**Fig. 3:**
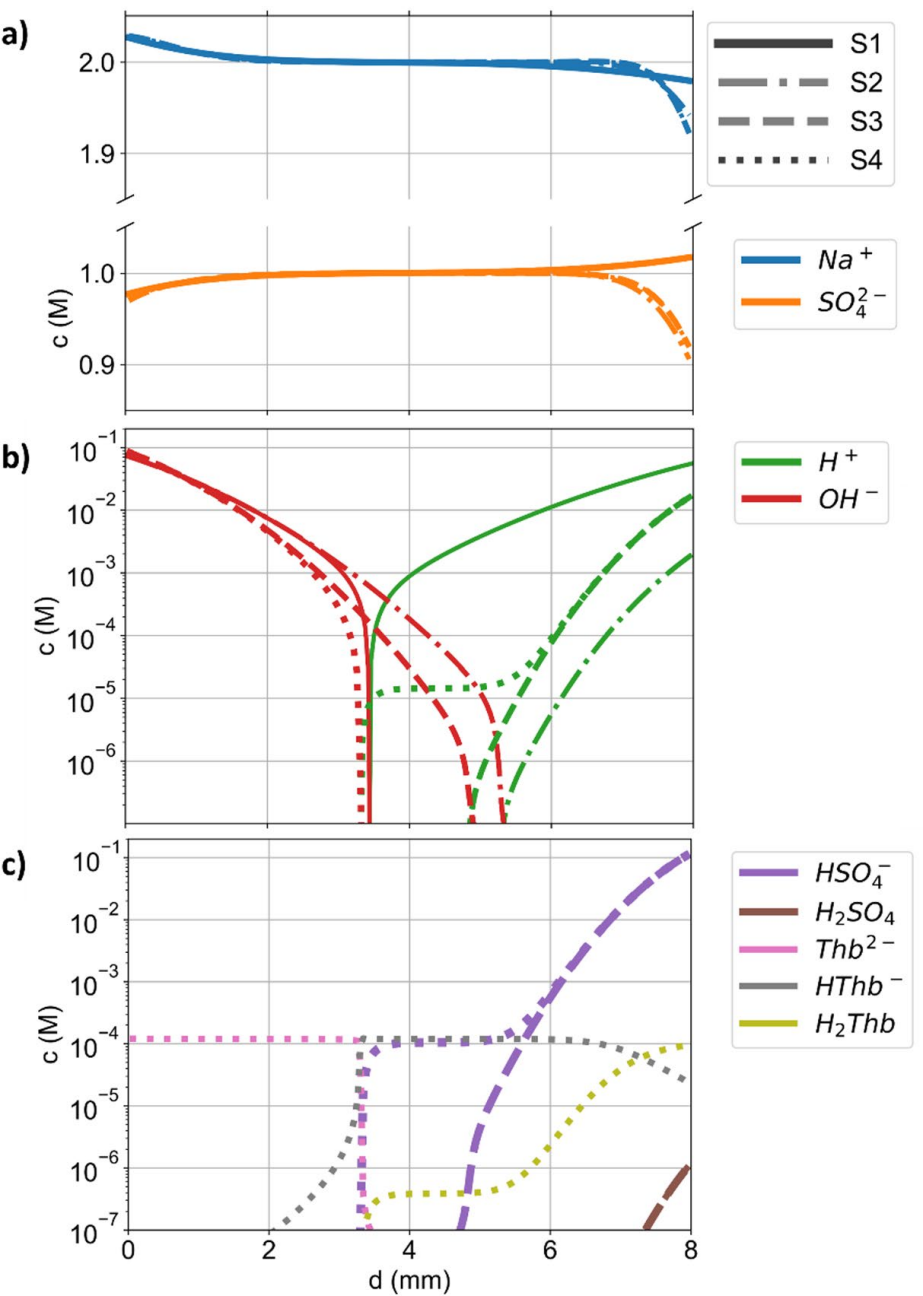
Simulated concentrations c in (**a**) a linear scale and (**b**) and (**c**) a logarithmic scale of selected species in solution for S1, S2, S3, and S4 after 200 s as a function of the distance d to the cathode. The linestyles for S1 to S4 are displayed in a).

Figure 3 shows the simulated concentration profiles for S1 to S4 after 200 s (where S4 supposedly represents the experimental data best). In Fig. 3a, the concentrations of Na^+^ and SO_4_^2−^ are shown. Na^+^ is transported towards the cathode and away from the anode. The sulphate ions are transported away from the cathode and towards the anode, as visible for S1. However, due to the homogeneous reactions considered in S2 to S4, the concentration of sulphate decreases below its initial value of 1 M close to the anode. Figure 3b shows the concentrations of H^+^, OH^−^, while Fig. 3c shows HSO_4_^−^,H_2_SO_4_, Thb^2−^, HThb^−^, and H_2_Thb in a logarithmic scale. For S1, steep concentration gradients for H^+^ and OH^−^ occur at $\:d\:=\:3.4$ mm due to the neutralization (pH 7) reaction of H^+^ and OH^−^ (forming water and thus displaying a sink for the respective ions). For S2 and S3 the H^+^-profiles intercept the OH^−^-profiles at $\:d=5.3$ and $\:d=4.8\:$mm, respectively, also displaying the point of pH 7. Hence, the H^+^ transport towards the cathode with S3 is faster than that with S2 (from *d* = 6 to 8 mm), although the magnitudes of the transport properties are reduced due to the ion-ion interactions. This faster effective transport results from including ionic activity coefficients in S3, which shift the equilibrium towards the dissociated state. In S4, the line of OH^−^ intercept with H^+^ and HSO_4_^−^ at roughly $\:d=3.2$ mm, respectively. Between 3.3 and 5 mm, the modelled concentration of H^+^ and HSO_4_^−^ in S4 is roughly constant (the concentrations are basically unchanged since the beginning of the experiment) due to the buffering effects of thymol blue slowing down the propagation of the alkaline phase. Compared to S3, the electrolyte in S4 is more acidic due to the dissociation of thymol blue, which further decreases the propagation velocity of the transition zone. For S3 and S4, molecular H_2_SO_4_ forms with concentrations > 10^−7^ M at the anode, which is not the case for S1 and S2. In Fig. 3c, the sharp transition from Thb^2−^ to HThb^−^ is visible. The location of this transition is close to the neutral pH (H^+^ and OH^−^ intercept) visible in Fig. 3c. The second transition, from HThb^−^ to H_2_Thb, spans a wider distance than the first one as the transition appears almost simultaneously to the reaction from sulfate to bisulfate (pK_s_ values of 1.7 and 1.9, respectively).

### Ion transport in buffered electrolytes

**Fig. 4:**
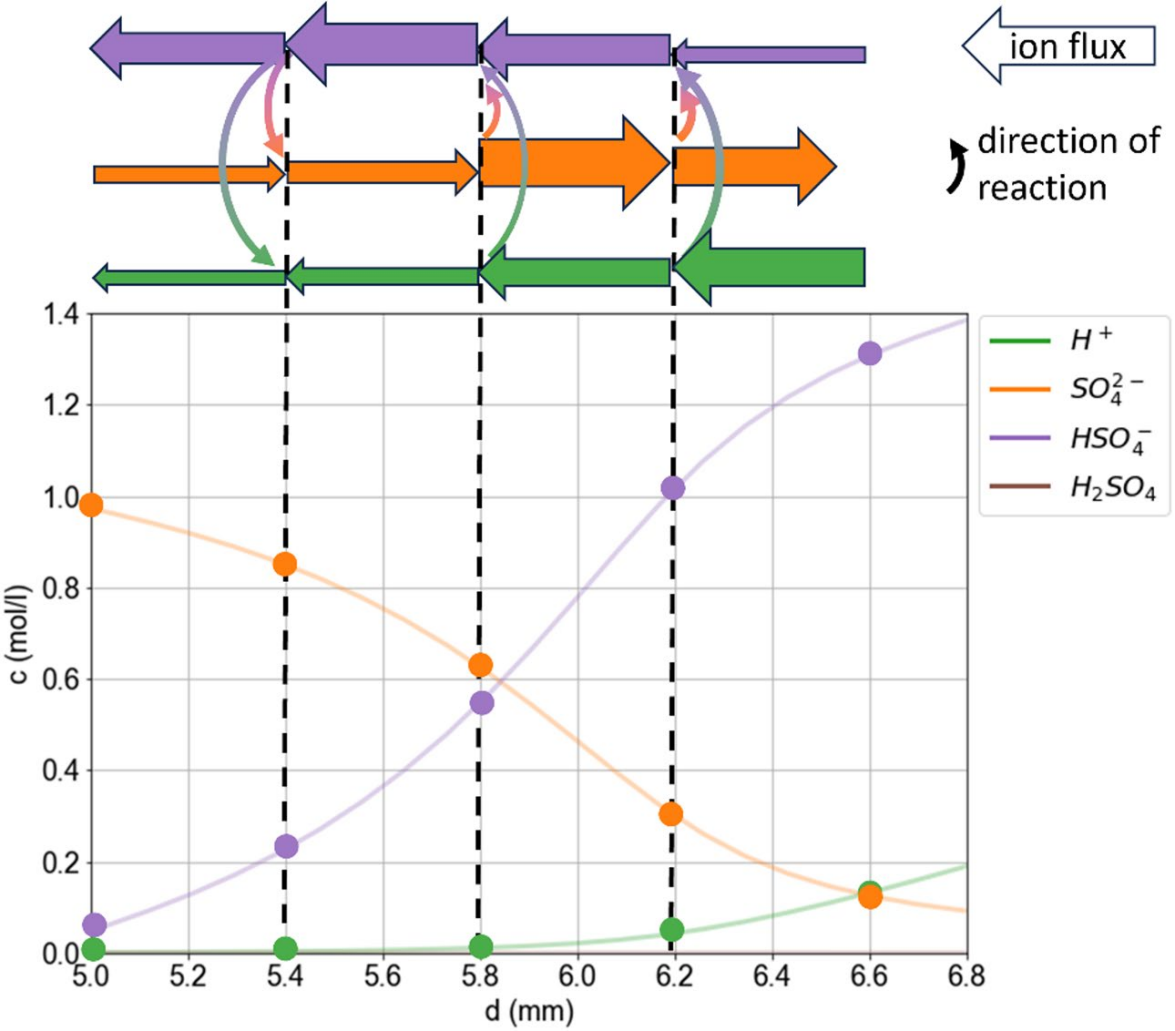
Illustration of the ionic fluxes (top) and concentrations c (bottom) simulated with S3. The widths of the large arrows qualitatively indicate the magnitude of the flux. Similarly, the rate and direction of the homogeneous reaction ($\:{H}^{+}+S{O}_{4}^{2-}\:\rightleftharpoons\:\:HS{O}_{4}^{-}\:$) are indicated by the size and direction of the curved arrows, respectively. The dots in the lower graph indicate the discrete concentrations in the simulation that are subjected to the fluxes. The lines illustrate the continuous concentration profiles (as a guide to the eye). Due to the equilibrium reactions, the effective fluxes of all species involved are reduced.

Figure 4 illustrates a schematic representation of the ion fluxes in a 1 M Na_2_SO_4_ solution (simulated with S3) for a current density of 10 mA/cm^2^ after 300 s. The fluxes and reactions are shown for discrete locations, representing discrete elements of the simulation. At the anode, H^+^ ions are released and transported to the cathode (left). Sulphate ions are transported towards the anode (illustrated by the large arrows), where they partly react with protons to form bisulphate and molecular sulfuric acid. Bisulphate and molecular sulfuric acid are both transported towards the cathode (due to their concentration gradient and a comparably small electric field). Close to the transition zone, the protons react with hydroxide ions to form water which shifts the reaction equilibrium to the left-hand side of the reaction, forming protons and sulphate ions from bisulphate. Hence, the flux of bisulphate increases due to the emerging concentration gradient. However, since the mobility of the bisulphate is much smaller than that of protons (roughly a factor of 9 at infinite dilution), the effective ion transport of protons is reduced. Similarly, the effective transport of sulphate is reduced because the bisulphate and molecular sulfuric acid transport sulphate towards the cathode instead of the anode.

### Parameter variation

**Fig. 5:**
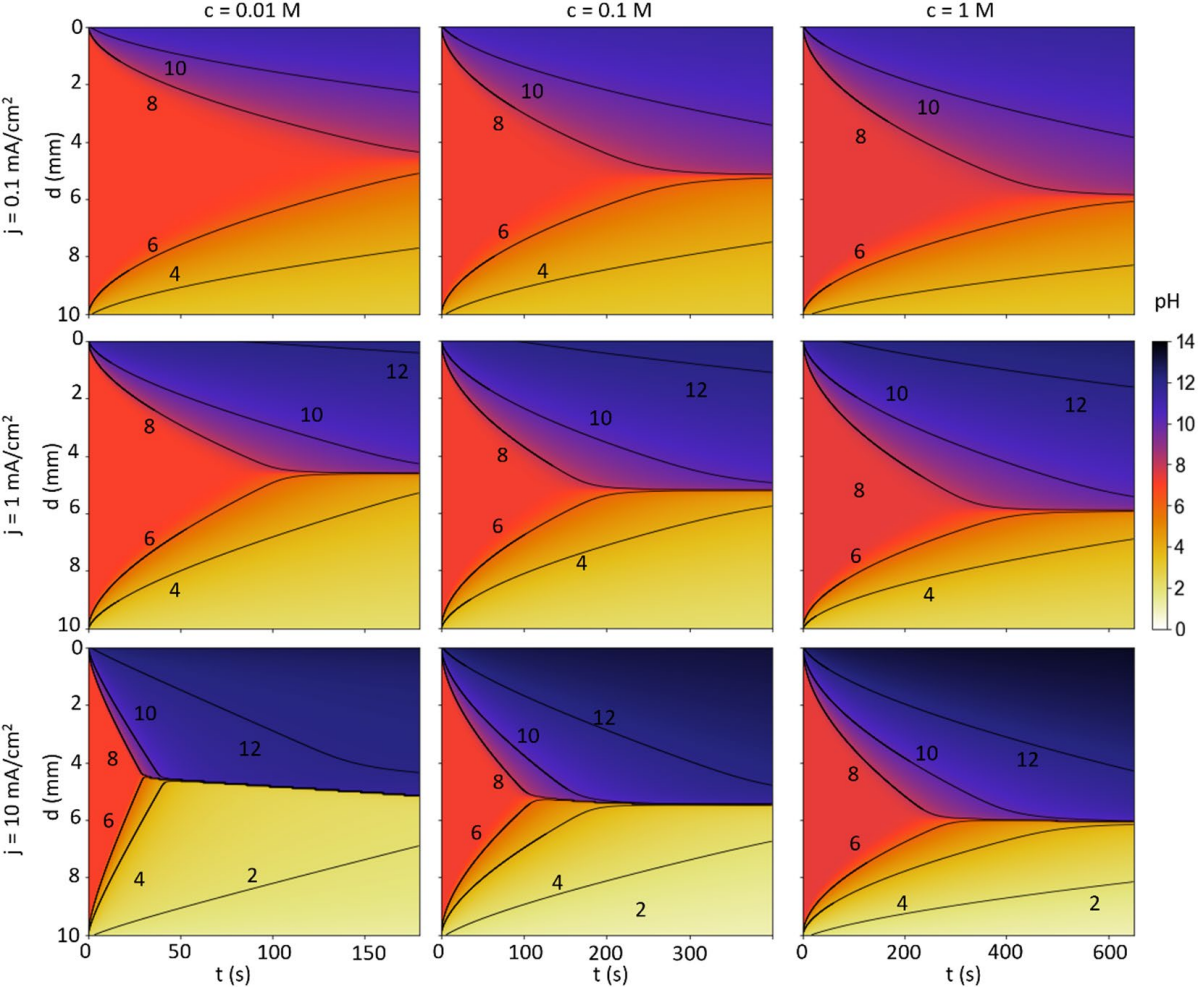
Colormaps of the modelled pH over time for the variation of the initial Na_2_SO_4_ concentration c and the current density for a distance between the electrodes 10 mm. With increasing current density and decreasing concentration, the pH gradients become steeper and the pH values more extreme. The concentration significantly affects the location of the neutral area, which is a result of the reduced effective transport of H^+^ towards the cathode.

Thus far, the pH gradient was considered only for one case with a specific electrolyte concentration and current density. To understand the effect of these parameters on the ion transport and resulting pH gradients, Fig. 5 shows a parameter variation of the current density ($\:0.1,\:1,$ and $\:10\:$mA/cm^2^) and the initial Na_2_SO_4_ concentration ($\:0.01,\:0.1,\:$ and $\:1$ M) for an electrode distance of $\:10$ mm using simulation S3. The graphed simulation times (180 s, 400 s, 650 s) for the different concentrations were chosen such that at $\:j=1$ mA/cm^2^ the contour lines of pH values 6 and 8 (near-neutral pH) agree within 0.2 mm after 75% of the simulation time.

In all simulations, the spatial zone with near-neutral pH values between 6 and 8 shrinks over time as H^+^ and OH^−^ produced at the electrodes penetrate into the bulk electrolyte. Towards higher current densities, the near-neutral zone shrinks faster, as the flux carried by the electromigration increases. Yet, a 100-fold in the current density causes just an approximately 3-times faster shrinkage of the near-neutral zone as diffusion displays the bottleneck for approaching the steady states of the concentration profiles. Supplementary Fig. 2.6 illustrates this diffusion-limited transport, with a nearly linear relation of the pH contour lines as a function of the square-root time, as expected from an idealized diffusion process. Moreover, Supplementary Fig. 2.7 shows an unphysical yet didactically expressive variation of the diffusion coefficients, emphasising that diffusion displays the time-limiting process for the evolution of the concentration profiles.

Towards larger concentrations, the pH gradient becomes more distinct, as the total number of H^+^ and OH⁻ ions that can accumulate (and thus the minimum and maximum pH, respectively) are constrained by the amount of supporting electrolyte due to the electroneutrality. Increasing the initial Na_2_SO_4_ concentration also shifts the final location of the near-neutral area shifts towards the anode. Higher sulphate concentrations shift the reaction equilibrium of the homogeneous reaction with H^+^ towards bisulphate and molecular sulfuric acid (being basically negligible at the pH values in the discussed systems), decreasing the H^+^ concentration. Hence, the concentrations and concentration gradients of H^+^ at the anode have a smaller magnitude than the ones of OH^−^ at the cathode. Therefore, the flux of OH^−^ is larger than that of H^+^, resulting in a more spatially expanded alkaline than acidic zone. Initially, the cells with higher concentrations show a slightly more alkaline pH due to the buffer reactions of the sulphate.

Figure 5 shows the complex interference of different parameters on pH profiles using sulfate as an exemplary buffer. pH indicators like thymol blue are buffers, too. Hence, the transient propagation of pH fronts depends on the concentration and pK_a_ values of the pH indicator used. Importantly, the buffer effects that slow down the propagation of pH fronts are always tied to the transition zone (so the exact region used for the measurement) because the optical change is a consequence of the buffer reaction. Increasing the pH indicator concentration, will increase the artefacts of the pH indicator on the measurement.

### Comparison to literature results

As discussed in the “Introduction”, natural convection and gas bubbles typically affect the ion transport in electrochemical cells. In this work, these artefacts were intentionally minimised by means of the gas-bubble-free and horizontal cell design, which purposely was designed to represent a one-dimensional ion transport without significant contributions of convection. In the literature^25–27^, less idealised reactors were used to evaluate ion transport and pH simulations, where convective mixing interacted with electromigration and diffusion. Thus, such experimental data displayed an unsolid starting point to develop simple one-dimensional transport models (which cannot account for the convection), explaining the large reported differences between computed and measured pH gradients^27–29^. Moreover, in few of these works the effects of pH indicators^27,33^ and dissolved CO_2_ were taken into consideration, adding to the uncertainty of the modelled data. By including these influences into the presented simulation, major improvements of the precision of pH predictions were achieved.

In this study, the concentration dependence of transport properties (diffusion coefficients, conductivities, and transference numbers), ionic activity coefficients, and chemical equilibria were considered. However, these physicochemical electrolyte properties (PCEPs) were considered as constant in literature models for pH gradients^27^ mostly using infinite dilution data instead. In Figs. 1, 2 and 3, the impact of the PCEPs on the ion transport is showcased by the comparison between simulation S2 and S3. Especially, the concentration-dependence of chemical equilibrium constants^39^ drastically affects concentrations in buffered electrolytes, affecting the effective transport of all ions in solution as well as the pH values. Consequently, neglected concentration dependencies of PCEPs partly explain the origin of imprecise simulations for predicting pH values^22,27,30–36^. These results underline the previously addressed importance of PCEPs for precise ion transport modelling^16,40,41^ which is here also emphasised for modelling pH gradients.

Concentration-dependent data for PCEPs of multi-ion electrolytes are rarely available and require considerable effort to be measured^15^ as they are affected by ion-ion interactions. As an alternative, PCEPs can be modelled using physicochemical models. However, to date, physicochemical models for PCEPs, which are often based on the Debye-Hückel theory or the MSA, suffer from limited predictive capabilities, especially for multi-ion electrolytes and at practically relevant concentrations (> 1 M)^16,17^. For example, modelling the activity coefficients and transport properties of aqueous sulfuric acid remains a debated challenge in the literature^42–44^. Hence, modelling mixtures that include H_2_SO_4_ include this unresolved challenge and add further uncertainties to the simulations. Nevertheless, in this study, using the MSA to estimate PCEPs has provided the fundament for substantial improvements of pH predictions.

For non-ideal electrochemical reactors, such as CO_2_ or water electrolysers, this study lays a solid foundation for modelling the aqueous phase accurately. For example, the gas bubble formation in CO_2_ electrolysers due to pH shifts can be quantified more precisely. Furthermore, the activity of the species and the pH in close proximity to the electrodes can be calculated more accurately, enabling more accurate insights into reaction pathways and corrosion mechanisms.

## Conclusions

In this study, the effects of pH buffers on ion transport and the development of local pH values were examined in a combined experimental and simulation study. An electrochemical reactor was developed to optically investigate the pH of the electrolyte in situ. The transition zone of thymol blue between pH 8.0 and 9.6 was used to evaluate the pH optically. The experimental data were compared to ion transport simulations that incorporate models for the activity coefficients and transport properties using the Mean Spherical Approximation and the data at infinite dilution. Using transport properties at infinite dilution for the simulations, the simulated propagation of the transition zone was faster than that observed in the experiments. By including the effects of ionic strength dependent electrolyte transport properties, homogeneous reactions, and the buffering capacity of the pH indicator on the ion transport, an accurate simulation of the measured transition zone propagation was achieved. Importantly, the buffering capacity of pH indicators affects the effective ion transport of H^+^ and OH^−^, showing their invasive influence on the measurements (contrary to previous literature statements). For the experimental evaluation, a vertical cell design with stacked electrolyte densities minimised natural convection by density gradients. For application of the ion-transport simulation to non-idealised electrochemical cells, convection and bubble formation in three-dimensions must be addressed in future works.

## Methods

### Experimental

**Fig. 6:**
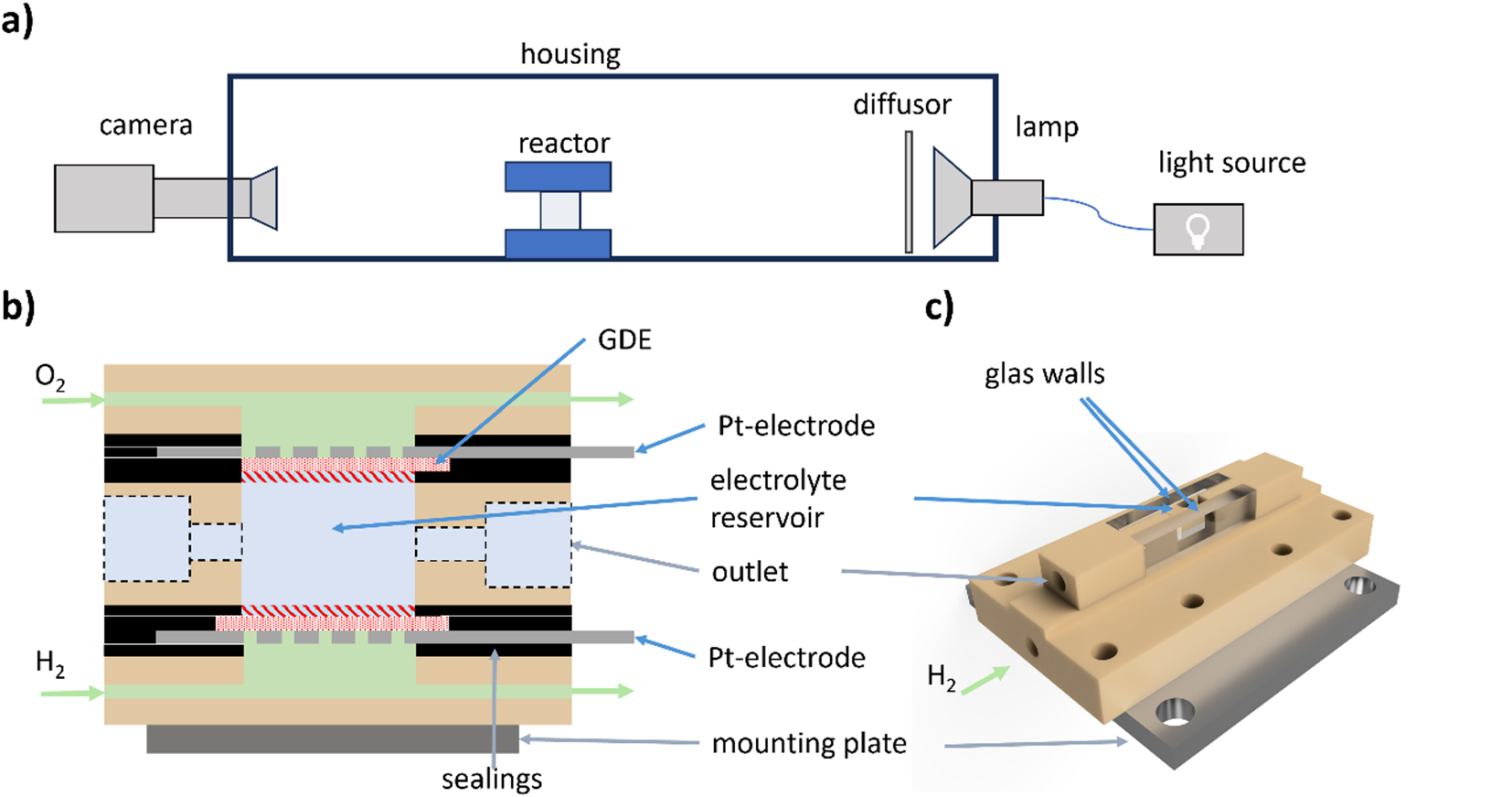
Experimental setup for optical pH measurements. (**a**) Schematic overview of the whole setup. (**b**) Schematic cross section of the reaction. The red-shaded area indicates the area which is optically not observable. (**c**) 3D visualization of the optical cell.

To enable locally resolved optical in situ measurements of pH values across the electrolyte, an electrochemical cell with glass windows was designed. The experimental setup for the optical measurements is illustrated in Fig. 6a. The electrochemical cell is placed between a camera and a lamp (like the setup reported in the literature^37,45^. The light coming from the light source is diffused by two PTFE sheets (0.3 mm) before going through the reactor. To shield the setup from external light sources, it is covered by a plastic housing. Figure 6b shows a schematic cross section of the optical reactor. At the cathode, pure oxygen is reduced, while at the anode hydrogen is oxidised. The fuel cell setup avoids the formation of bubbles that would lead to convection and blockage of electrode surface area. Aiming to avoid natural convection due to density gradients^29^ the gas-diffusion-electrodes (GDE, Freudenberg H23C3 with a Pt-loading of 1.28 mg/cm^2^) are placed horizontally. Figure 6c shows a 3D visualization of the electrochemical cell (without sealings). The sealings are made from EPDM sheets with a thickness of 0.5 mm. The optically observable region has a height of 7 mm and a width of 10 mm. The distance between the GDEs is 8 mm. A perforated Pt sheet serves as the current collector. The electrolyte is confined between two quartz glass plates that are arranged in parallel with a distance of 3 mm.

A 1 M solution made of Na_2_SO_4_ (anhydrous, 99% from ThermoFisher, Lot: 10227817) and deionised water (18 MΩ) serves as supporting electrolyte. For the pH evaluation, thymol blue (ACS grade from ThermoFisher, Lot: Z11G065) is used as pH indicator. Thymol blue showed no signs of degradation in preliminary long-term tests at Pt-electrodes. The employed concentration of thymol blue was 0.12 mM. The conductivity of the pH-indicator species is negligible compared to the excess of supporting electrolyte.

### pH evaluation

For the evaluation of the pH, the colour transition of thymol blue from yellow (pH from 2.8 to 8) to blue (pH > 9.6) was evaluated. In the Supporting Information (Supplementary Figure S2.1), an exemplary image is shown, which illustrates the area used for the evaluation of the optical data. The distance *d* between location of the pH shift and the cathode was evaluated using an automated procedure implemented in Python. For the evaluation of *d*, the optical data (TIFF images) are averaged horizontally within the specified rectangle.

### Simulation

**Fig. 7:**
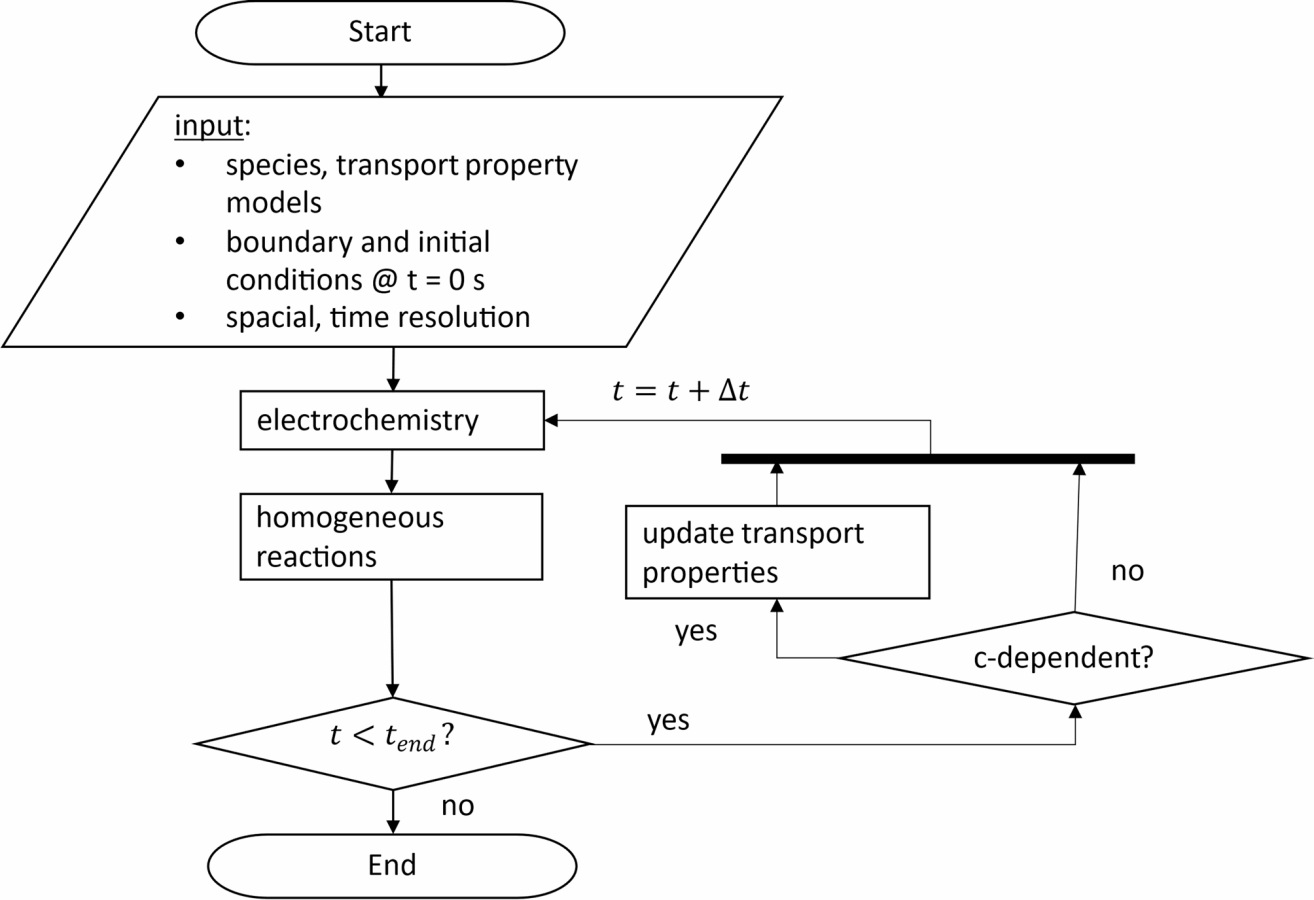
Flow chart of the calculation procedure used for the ion transport simulation.

The ion transport model is implemented in a one-dimensional framework in Python. The procedure for the simulations of the transient ion transport is illustrated in (Fig. 7). First, the initial and boundary conditions such as the concentration of the supporting electrolyte ($\:{c}_{N{a}_{2}S{O}_{4}}=1\:\text{M}$), the electrode distance ($\:{d}_{0}=8\:\text{m}\text{m}$), number of cells ($\:n=200$), time increment ($\:{\Delta\:}t=0.001\:\text{s}$), current density ($\:i=3.33\:\text{m}\text{A}/\text{c}{\text{m}}^{2}$), electrode reactions and so forth are specified. For the time-loop, a Forward-Euler scheme is applied. To account for the electrode reactions, the change in concentrations of H^+^ and OH^−^ are calculated from Faraday’s law for the cells at the electrodes based on the current density and time increment. After the electrochemical transport, the homogeneous reactions are calculated, partly affecting the concentration-profiles of the ion species in the electrolyte. The activity coefficients and transport properties are either calculated based on the MSA^46^ or the data at infinite dilution is used.

The transport model (being part of the “electrochemistry” module in Fig. 7) developed here is based on the modified Nernst-Planck equation which uses concentration-dependent diffusion coefficients and conductivities (instead of the data at infinite dilution)^18^. The calculated properties (MSA) account for the concentration-dependent ion-ion interactions but were derived for pure diffusion and pure migration (as discussed below). Strictly, using Stefan-Maxwell-type transport models^47,48^ (like Onsager’s^49^ and Newman’s^15,50^ models) that explicitly account for the mutual diffusivity (or friction) of all species with respect to each other would be more accurate. However, at the used concentrations, the respective diffusion coefficients between ions are typically much smaller than the diffusion coefficients between an ion and the solvent (see for example H_2_SO_4_^51^). Therefore, at the experimental concentrations used here, using the (modified) Nernst-Planck equation is justified. Furthermore, the one-dimensionality simplifies the electroneutrality enforcementfor which the previously reported analytical “method M4” from our previous study^18^ is used. The applied method takes diffusion potentials for the calculation of the potential gradients into account, which automatically ensures electroneutrality. In the one-dimensional case, the calculation of diffusion potentials is straightforward because the local current density is always known and constant.

### Parameterization

The concentration-dependent parametrization is based on the MSA for multi-ion electrolytes^37^ which uses ionic radii, equilibrium constants (K_a_ or pK_a_ values), ionic parameters for the viscosity and limiting equivalent conductivities as input parameters. All employed equations are given in the Supplementary Information. In the literature, it is well established that the ionic radii serve as fitting parameters to optimize the fit between the model and measurement data^17^. However, different ionic radii for a specific ion (for example SO_4_^2−^) are necessary to optimize the fit for different salts (for example H_2_SO_4_, Na_2_SO_4_). Furthermore, the optimal ionic radii may differ for different physicochemical properties^16^. Here, one set of parameters for all ionic species is used, which shows a good compromise between activity coefficients, viscosities, mutual diffusion coefficients, and conductivities. All parameters used are supplied in the Supplementary Information.

**Fig. 8:**
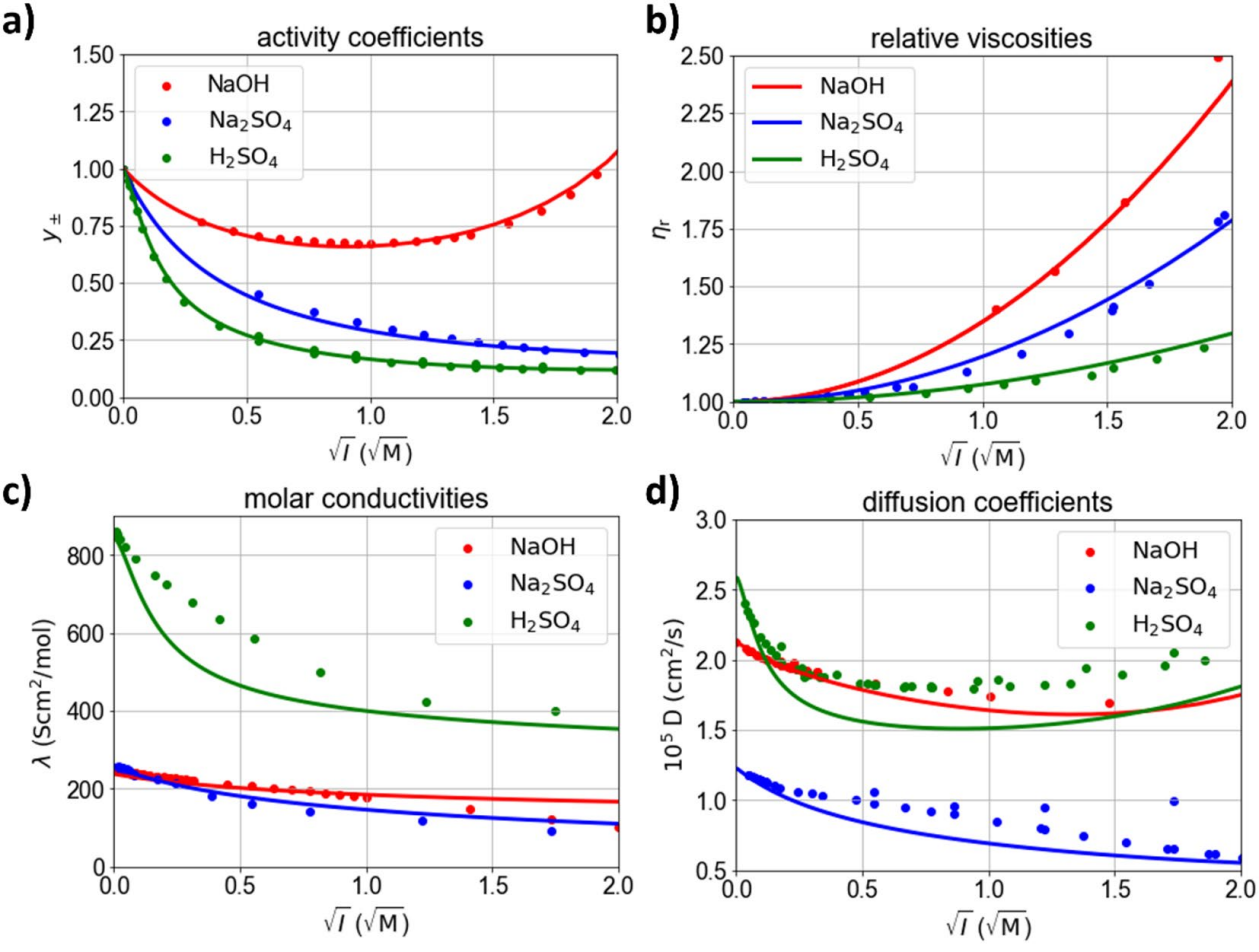
Comparison of electrolyte properties including experimental data from the literature (dots) and the MSA-model (lines) for NaOH, Na_2_SO_4_, and H_2_SO_4_. (**a**) Activity coefficients^14,52^. (**b**) Viscosities^14,53^. (**c**) Molar conductivity^14^. (**d**) Mutual diffusion coefficients^14,44,54^.

Figure 8 shows the modelled electrolyte properties and measured data from the literature for the binary species that appear in the considered electrochemical system (NaOH, Na_2_SO_4_, H_2_SO_4_) as a function of the ionic strength *I* (assuming complete dissociation for the scale), respectively. For NaOH and Na_2_SO_4_, complete dissociation is assumed in the considered concentration range. For H_2_SO_4_, homogeneous reactions are implemented based on the pK_a_ values of the first ($\:{\text{H}}^{+}+\text{H}\text{S}{\text{O}}_{4}^{-}\rightleftharpoons\:{\text{H}}_{2}\text{S}{\text{O}}_{4}$: pK_a,1_ = -3) and second ($\:{\text{H}}^{+}+\text{S}{\text{O}}_{4}^{2-}\rightleftharpoons\:\text{H}\text{S}{\text{O}}_{4}^{-}$: pK_a,2_ = 1.9) association reaction. Figure 8a shows the mean ionic activity coefficient $\:{y}_{\pm\:}$ (on the molar scale). The homogeneous reactions of H_2_SO_4_ account for the large difference between the activity coefficients of Na_2_SO_4_ and H_2_SO_4_. The good match between measured and simulated activity coefficients justifies the assumptions regarding the homogeneous reactions. Figure 8b shows modelled viscosities. Figure 8c and d show the conductivity and the mutual diffusion coefficient for the three considered binary electrolytes, respectively^44^. Except for the conductivity of H_2_SO_4_, all transport properties and the activity coefficients of the binary electrolytes were modelled accurately based on single ion properties.

## Supplementary Information

Below is the link to the electronic supplementary material.

Supplementary Material 1

## Data Availability

The data are available under: 10.26165/JUELICH-DATA/XNE45K.

## References

[1] Alves Goulart, D. & Dutra Pereira, R. Autonomous pH control by reinforcement learning for electroplating industry wastewater. *Comput. Chem. Eng.* **140**, 106909. 10.1016/j.compchemeng.2020.106909 (2020).

[2] Passos, A. C. M. et al. Effect of pH and current density on the physical properties of Cobalt obtained by electrowinning from sulfate solutions. *Miner. Eng.* **211**, 108697. 10.1016/j.mineng.2024.108697 (2024).

[3] Chen, Y. et al. Situ visualization of ion transport processes in aqueous batteries. *ACS Appl. Mater. Interfaces*. **16**, 42321–42331. 10.1021/acsami.4c09788 (2024). 39088694 10.1021/acsami.4c09788

[4] Karlsson, R. K. B. & Cornell, A. Selectivity between oxygen and Chlorine evolution in the Chlor-Alkali and chlorate processes. *Chem. Rev.* **116**, 2982–3028. 10.1021/acs.chemrev.5b00389 (2016). 26879761 10.1021/acs.chemrev.5b00389

[5] Zhang, Z. et al. pH matters when reducing CO 2 in an electrochemical flow cell. *ACS Energy Lett.* **5**, 3101–3107. 10.1021/acsenergylett.0c01606 (2020).

[6] Zhang, F. & Co, A. C. Direct evidence of local pH change and the role of alkali cation during CO2 electroreduction in aqueous media. *Angew. Chem. Int. Ed. Engl.* **59**, 1674–1681. 10.1002/anie.201912637 (2020). 31721382 10.1002/anie.201912637

[7] Monteiro, M. C. O. et al. Time-resolved local pH measurements during CO_2_ reduction using scanning electrochemical microscopy: Buffering and tip effects. *JACS Au* **1**, 1915 (1924). 10.1021/jacsau.1c00289 (2021). 10.1021/jacsau.1c00289 PMC8611793 34849509

[8] Varela, A. S., Kroschel, M., Reier, T. & Strasser, P. Controlling the selectivity of CO2 electroreduction on copper: the effect of the electrolyte concentration and the importance of the local pH. *Catal. Today*. **260**, 8–13. 10.1016/j.cattod.2015.06.009 (2016).

[9] Naito, T., Shinagawa, T., Nishimoto, T. & Takanabe, K. Water electrolysis in saturated phosphate buffer at neutral pH. *ChemSusChem* **13**, 5921–5933. 10.1002/cssc.202001886 (2020). 32875653 10.1002/cssc.202001886 PMC7756658

[10] Paepe, J. et al. Electrochemical in situ pH control enables Chemical-Free full urine nitrification with concomitant nitrate extraction. *Environ. Sci. Technol.* **55**, 8287–8298. 10.1021/acs.est.1c00041 (2021). 34086451 10.1021/acs.est.1c00041

[11] Slesinski, A., Sroka, S., Fic, K., Frackowiak, E. & Menzel, J. Operando monitoring of local pH value changes at the carbon electrode surface in neutral Sulfate-Based aqueous electrochemical capacitors. *ACS Appl. Mater. Interfaces*. **14**, 37782–37792. 10.1021/acsami.2c09920 (2022). 35946232 10.1021/acsami.2c09920 PMC9412948

[12] Bollella, P., Melman, A. & Katz, E. Operando local pH mapping of electrochemical and bioelectrochemical reactions occurring at an electrode surface: effect of the buffer concentration. *ChemElectroChem* **8**, 3923–3935. 10.1002/celc.202101141 (2021).

[13] Kas, R., Kortlever, R., Yılmaz, H., Koper, M. T. M. & Mul, G. Manipulating the hydrocarbon selectivity of copper nanoparticles in CO 2 electroreduction by process conditions. *ChemElectroChem* **2**, 354–358. 10.1002/celc.201402373 (2015).

[14] Thomas, W., Chapman & Newman, J. *A compilation of selected thermodynamic and transport properties of binary electrolytes in aqueous solution* (1968).

[15] Newman, J. S. & Balsara, N. P. *Electrochemical Systems* (John Wiley & Sons, Inc, 2021).

[16] Janotta, B., Schalenbach, M., Tempel, H. & Eichel, R. A. Fitting ambiguities mask deficiencies of the Debye-Hückel theory: revealing inconsistencies of the Poisson-Boltzmann framework and permittivity. *Phys. Chem. Chem. Phys.* **27**, 7703–7715. 10.1039/d5cp00646e (2025). 40151984 10.1039/d5cp00646e

[17] Villard, A., Bernard, O. & Dufrêche, J. F. Non-additivity of ionic radii in electrolyte solutions: hofmeister effect on mixtures modeled by an associated MSA model. *J. Mol. Liq.* **270**, 30–39. 10.1016/j.molliq.2018.01.125 (2018).

[18] Janotta, B., Schalenbach, M., Tempel, H. & Eichel, R. A. An assessment of electroneutrality implementations for accurate electrochemical ion transport models. *Electrochim. Acta*. **508**, 145280. 10.1016/j.electacta.2024.145280 (2024).

[19] Katsounaros, I. et al. The effective surface pH during reactions at the solid–liquid interface. *Electrochem. Commun.* **13**, 634–637. 10.1016/j.elecom.2011.03.032 (2011).

[20] Monteiro, M. C. & Koper, M. T. Measuring local pH in electrochemistry. *Curr. Opin. Electrochem.* **25**, 100649. 10.1016/j.coelec.2020.100649 (2021).

[21] Schatz, M., Jovanovic, S., Eichel, R. A. & Granwehr, J. Quantifying local pH changes in carbonate electrolyte during copper-catalysed formula: see text electroreduction using in Operando formula: see text NMR. *Sci. Rep.* **12**, 8274. 10.1038/s41598-022-12264-8 (2022). 35585102 10.1038/s41598-022-12264-8 PMC9117298

[22] Critelli, R. A., Bertotti, M. & Torresi, R. M. Probe effects on concentration profiles in the diffusion layer: computational modeling and near-surface pH measurements using microelectrodes. *Electrochim. Acta*. **292**, 511–521. 10.1016/j.electacta.2018.09.157 (2018).

[23] Yin, F. & Liu, H. The j–pH diagram of interfacial reactions involving H + and OH–. *J. Energy Chem.* **50**, 339–343. 10.1016/j.jechem.2020.03.078 (2020).

[24] Yin, F. et al. Acid–base transport model depicting the dynamic pH response of interfacial reactions. *AIChE J.* **68**10.1002/aic.17669 (2022).

[25] Steinegger, A., Wolfbeis, O. S. & Borisov, S. M. Optical sensing and imaging of pH values: spectroscopies, materials, and applications. *Chem. Rev.* **120**, 12357–12489. 10.1021/acs.chemrev.0c00451 (2020). 33147405 10.1021/acs.chemrev.0c00451 PMC7705895

[26] Steininger, F. & Koren, K. Situ pH modulation for enhanced chemical sensing: strategies and applications. *Anal. Sens.* **4**10.1002/anse.202400013 (2024).

[27] Pande, N. et al. Electrochemically induced pH change: Time-Resolved confocal fluorescence microscopy measurements and comparison with numerical model. *J. Phys. Chem. Lett.* **11**, 7042–7048. 10.1021/acs.jpclett.0c01575 (2020). 32787336 10.1021/acs.jpclett.0c01575 PMC7476033

[28] Yang, K., Kas, R. & Smith, W. A. In situ infrared spectroscopy reveals persistent alkalinity near electrode surfaces during CO2 electroreduction. *J. Am. Chem. Soc.* **141**, 15891–15900. 10.1021/jacs.9b07000 (2019). 31523949 10.1021/jacs.9b07000 PMC6788196

[29] Fuladpanjeh-Hojaghan, B. et al. In-Operando mapping of pH distribution in electrochemical processes. *Angew. Chem. Int. Ed. Engl.* **58**, 16815–16819. 10.1002/anie.201909238 (2019). 31538391 10.1002/anie.201909238

[30] Cannan, S., Douglas Macklam, I. & Unwin, P. R. Three-dimensional imaging of proton gradients at microelectrode surfaces using confocal laser scanning microscopy. *Electrochem. Commun.* **4**, 886–892. 10.1016/S1388-2481(02)00482-4 (2002).

[31] Stepan, T. et al. Effect of nanoparticle size on the near-surface pH-distribution in aqueous and carbonate buffered solutions. *Electrochim. Acta*. **409**, 139923. 10.1016/j.electacta.2022.139923 (2022).

[32] Leenheer, A. J. & Atwater, H. A. Imaging Water-Splitting electrocatalysts with pH-Sensing confocal fluorescence microscopy. *J. Electrochem. Soc.* **159**10.1149/2.022209jes (2012).

[33] Rudd, N. C. et al. Fluorescence confocal laser scanning microscopy as a probe of pH gradients in electrode reactions and surface activity. *Anal. Chem.* **77**, 6205–6217. 10.1021/ac050800y (2005). 16194080 10.1021/ac050800y

[34] Carneiro-Neto, E. B., Lopes, M. C. & Pereira, E. C. Simulation of interfacial pH changes during hydrogen evolution reaction. *J. Electroanal. Chem.* **765**, 92–99. 10.1016/j.jelechem.2015.09.029 (2016).

[35] Lu, X. et al. In situ observation of the pH gradient near the gas diffusion electrode of CO2 reduction in alkaline electrolyte. *J. Am. Chem. Soc.* **142**, 15438–15444. 10.1021/jacs.0c06779 (2020). 32692913 10.1021/jacs.0c06779

[36] Wu, J., Zheng, W. & Chen, Y. Factors affecting the cathode/electrolyte interfacial pH change during water reduction: A simulation study. *Int. J. Hydrog. Energy*. **2022**, 18597–18605 (2022).

[37] Schalenbach, M., Durmus, Y. E., Tempel, H., Kungl, H. & Eichel, R. A. Ion transport and limited currents in supporting electrolytes and ionic liquids. *Sci. Rep.* **12**, 6215. 10.1038/s41598-022-10183-2 (2022). 35418198 10.1038/s41598-022-10183-2 PMC9008042

[38] Blum, L. Mean spherical model for asymmetric electrolytes. *Mol. Phys.* **30**, 1529–1535. 10.1080/00268977500103051 (1975).

[39] Grenthe, I., Mompean, F., Spahiu, K. & Wanner, H.Guidelines for the extrapolation to zero ionic strength. TBD-2 (2013).

[40] Mistry, A. & Srinivasan, V. Do we need an accurate Understanding of transport in electrolytes? *Joule* **5**, 2773–2776. 10.1016/j.joule.2021.10.007 (2021).

[41] Wang, A. A. et al. Potentiometric MRI of a superconcentrated lithium electrolyte: testing the irreversible thermodynamics approach. *ACS Energy Lett.* **6**, 3086–3095. 10.1021/acsenergylett.1c01213 (2021). 34541321 10.1021/acsenergylett.1c01213 PMC8438662

[42] Fraenkel, D. Electrolytic nature of aqueous sulfuric acid. 1. Activity. *J. Phys. Chem.* **116**, 11662–11677. 10.1021/jp3060334 (2012). 10.1021/jp3060334 22924586

[43] Fraenkel, D. Electrolytic nature of aqueous sulfuric acid. 2. Acidity. *J. Phys. Chem.* **116**, 11678–11686. 10.1021/jp306042q (2012). 10.1021/jp306042q 22924595

[44] Leaist, D. G. Diffusion in aqueous solutions of sulfuric acid. *Can. J. Chem.* **62**, 1692–1697. 10.1139/v84-290 (1984).

[45] Schalenbach, M. et al. Ionic transport modeling for liquid electrolytes - Experimental evaluation by concentration gradients and limited currents. *Electrochem. Sci. Adv.* **3**10.1002/elsa.202100189 (2023).

[46] Dufrêche, J. F., Bernard, O. & Turq, P. Transport equations for concentrated electrolyte solutions: reference frame, mutual diffusion. *J. Chem. Phys.* **116**, 2085–2097. 10.1063/1.1427724 (2002).

[47] Maxwell, J. C. On the dynamical theory of gases. *Philosoph. Trans. R. Soc.* **1867**, 49–88 .

[48] Stefan, J. Über Das Gleichgewicht und bewegung, Insbesondere die diffusion von gasgemengen. *Sitzungsberichte Der Akademie Der Wissenschaften*. **1871**, 63–124 (1871).

[49] Onsager, L. Reciprocal relations in irreversible processes. I. *Phys. Rev.* **37**, 405–426. 10.1103/PhysRev.37.405 (1931).

[50] Newman, J., Bennion, D. & Tobias, C. W. Mass transfer in concentrated binary electrolytes. *Ber Bunsenges Phys. Chem.* **69**, 608–612. 10.1002/bbpc.19650690712 (1965).

[51] Umino, S. & Newman, J. Temperature dependence of the diffusion coefficient of sulfuric acid in water. *J. Electrochem. Soc.* **144**, 1302–1307. 10.1149/1.1837588 (1997).

[52] Wirth, H. E. Activity coefficients in sulphuric acid and sulphuric-Acid-Sodium-Sulphate mixtures. *Electrochim. Acta*. **1971**, 1345–1356 (1971).

[53] Robinson, R. A. & Stokes, R. H. *Electrolyte Solutions. Second Revised Edition* 2nd edn (Dover, 2002).

[54] Rard, J. A. & Miller, D. G. The mutual diffusion coefficients of Na2SO4 and MgSO4 at 25 C from Rayleigh interferometry. *J. Solut. Chem.* **8**, 755–766. 10.1007/BF00648779 (1979).

