# Supplementary material for: Buffering effects of supporting electrolytes on pH profiles in electrochemical cells
Source: Sci Rep. 2025 Sep 12;15:32458. doi: 10.1038/s41598-025-18219-z (PMC12432143; doi:10.1038/s41598-025-18219-z)
Supplement: Supplementary file 1 — Supplementary Material 1 [file 41598_2025_18219_MOESM1_ESM.pdf]

Supporting Information

for

Buffering effects of supporting electrolytes on pH profiles in electrochemical cells

by

Benjamin Janotta*[a], Maximilian Schalenbach*[a], Marcel Turiaux[a], Hermann Tempel[a], Rüdiger-A. Eichel[a,b,c]

[a]Fundamental Electrochemistry (IET-1), Institute of Energy Technologies, Forschungszentrum Jülich, Wilhelm-Johnen-Straße, 52425 Jülich, Germany

[b]Institute of Physical Chemistry, RWTH Aachen University, 52062 Aachen, Germany

[c]Faculty of Mechanical Engineering, RWTH Aachen University, Aachen, Germany

*Corresponding authors: b.janotta@fz-juelich.de, m.schalenbach@fz-juelich.de

1. Electrolyte Modelling

In this Chapter, the electrolyte model and the implementation using Python are briefly explained. Fig. 1.1 shows a flow chart for the ion transport simulations. The transport model (being part of the "electrochemistry" module in Fig. 1.1) developed here is based on the modified Nernst-Planck equation (Chapter 1.1) which uses concentration-dependent diffusion coefficients and conductivities (instead of the data at infinite dilution). The calculated properties (based on the Mean Spherical Approximation, Chapter 1.2) account for the concentration-dependent ion-ion interactions but were derived for pure diffusion and pure migration.

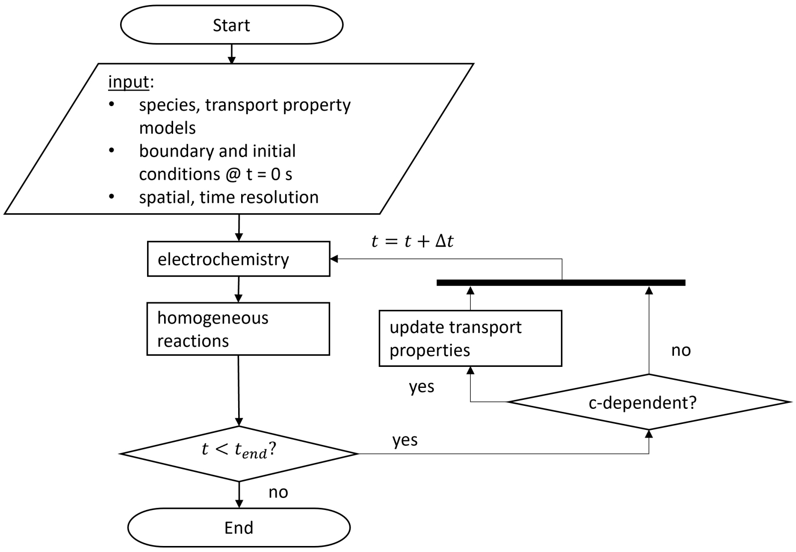

Figure 1.1: Flow chart of the calculation procedure used for the ion transport simulation.

1.1 Modified Nernst-Planck theory

In this work, the ionic flux $\boldsymbol{J}$ of a species is based on a modified Nernst-Planck theory [1–3]. The Nernst-Planck theory describes the ionic flux of a species as superposition of the contributions due to diffusion (chemical driving force) and migration (electrical driving force). The Nernst-Planck theory can be derived assuming that the flux of k is a linear response to a (small) gradient in electrochemical poten-

tial $\nabla\mu_k$:

$$\boldsymbol{J_k} = -u_k c_k \nabla\mu_k, \tag{1.1}$$

where u is the mobility and c is the concentration. The electrochemical potential may be described as a superposition of the chemical potential μ_k^* and the electrostatic potential Φ:

$$\mu_k = \mu_k^* + z_k F\Phi, \tag{1.2}$$

where z is the valency, and F denotes Faraday's constant. Furthermore, the chemical potential can be expressed as

$$\mu_k^* = RT\ln(a_k). \tag{1.3}$$

Inserting Eq. 1.2 into Eq. 1.1 and substituting the chemical potential with the activity (Eq. 1.3), leads to:

$$\begin{aligned}\boldsymbol{J_k} &= -u_k c_k \nabla(RT\ln(a_k) + z_k F\Phi), &(1.4)\\ &= -RTu_k c_k \nabla\ln(a_k) - u_k c_k z_k F\nabla\Phi, &(1.5)\\ &= -RTu_k c_k \frac{\nabla a_k}{a_k} - u_k c_k z_k F\nabla\Phi. &(1.6)\end{aligned}$$

Assuming an ideal solution ($a_k = c_k$) and using the Nernst-Einstein equation (Eq. 1.7):

$$D_k^0 = RTu_k^0, \tag{1.7}$$

where R is the ideal gas constant, T is the temperature and the superscript 0 denotes the value at infinite dilution, gives:

$$\boldsymbol{J_k} = -D_k^0\nabla c_k - u_k^0 c_k z_k F\nabla\Phi. \tag{1.8}$$

Eq. 1.8 displays the Nernst-Planck theory where the first term accounts for the diffusion (resembling Fick's first law of diffusion) and the second term accounts for the migration. The Nernst-Planck theory as well as the Nernst-Einstein equation are strictly only valid at infinite dilution.

In this work, the solution is assumed not to be ideal. Like in the Nernst-Planck theory, the ionic fluxes are calculated as a super-position of movement due to elec-

trical and chemical driving forces. To account for the concentration-dependence of the migration, the second term of Eq. 1.6 is rewritten in terms of ionic equivalent conductivities λ_k:

$$\boldsymbol{J}_k^{\text{migration}} = -\frac{\lambda_k c_k}{z_k F} \nabla \Phi. \tag{1.9}$$

In Eq. 1.9, the conductivities can be expressed as a function of concentrations. Similarly, by using the definition of activity coefficients y (on a molar scale):

$$a_k = c_k y_k, \tag{1.10}$$

to account for the concentration-dependence of the diffusion, the first term of Eq. 1.6 is rewritten:

$$\begin{aligned}
\boldsymbol{J}_k^{\text{diffusion}} &= -RT u_k c_k \frac{\nabla a_k}{a_k} && (1.11)\\
&= -D_k c_k \frac{\nabla (c_k y_k)}{c_k y_k} && (1.12)\\
&= -D_k \frac{\nabla c_k y_k + c_k \nabla y_k}{y_k} && (1.13)\\
&= -D_k \left(1 + \frac{d \ln y_k}{d \ln c_k}\right) \nabla c_k. && (1.14)
\end{aligned}$$

The terms inside the brackets in Eq. 1.14 are known as the thermodynamic factor, which describes the difference between diffusion in thermodynamically ideal and non-ideal solutions. Like the ionic conductivity, the diffusion coefficient D_k varies with concentration due to more interaction between ions.

1.2 Modelling Electrolyte Properties with the Mean Spherical Approximation

The thermodynamic and transport properties are modelled based on the Mean Spherical Approximation. For the derivation of the MSA, the reader is referred to the literature [4–6]. Here, the used equations are shown and their impact on the activity coefficients, and transport properties is presented.

1.2.1 Thermodynamics

Activity coefficients In the MSA, the activity coefficient of an ion k consists of two contributions, the electrostatic (el) contribution, and the hard sphere (HS) contribution:

$$\ln y_k = \ln y_k^{\mathrm{HS}} + \ln y_k^{\mathrm{el}}. \tag{1.15}$$

The hard sphere contribution can be calculated based on Eq. 1.16:

$$\ln y_k^{\mathrm{HS}} = -\Delta + F_1\sigma_k + F_2\sigma_k^2 + F_3\sigma_k^3 \tag{1.16}$$

where the auxiliary variables F_1, F_2, F_3 are defined by:

$$F1 = \frac{3X_2}{\Delta}, \tag{1.17}$$

$$F2 = \frac{3X_1}{\Delta} + \frac{3X_2^2}{\Delta^2 X3} + \frac{3X_2^2}{X_3^2}\ln\Delta, \tag{1.18}$$

$$F3 = \left(X_0 - \frac{X_2^3}{X_3^2}\right)\frac{1}{\Delta} + \frac{3X_1X_2 - X_2^3/X_3^2}{\Delta^2} + \frac{2X_2^3}{X_3\Delta^3} - 2\frac{2X_2^3}{X_3^3}\ln\Delta. \tag{1.19}$$

The X_n are geometric values:

$$X_n = \frac{\pi}{6}\prod_j c_j N_A \sigma_j^n \tag{1.20}$$

where N_A is Avogadro's constant and Δ is the volume fraction not occupied by ions ("free" dielectric continuum):

$$\Delta = 1 - \frac{\pi}{6}\prod_j c_j N_A \sigma_j^3. \tag{1.21}$$

The thermodynamic contribution of hard spheres was rigorously developed by Carnahan and Starling [7].

The electrostatic contribution y_k^{el} can be simplified to:

$$\ln y_k^{\mathrm{el}} = -\frac{e^2}{4\pi k_B T\varepsilon}\left(\frac{\Gamma z_k^2}{1+\Gamma\sigma_k} + \zeta\sigma_k\left(\frac{2z_k - \zeta\sigma_k^2}{1+\Gamma\sigma_k} + \frac{\zeta\sigma_k^2}{3}\right)\right), \tag{1.22}$$

where e is the elementary charge, and ζ is defined by:

$$\zeta = \frac{\pi}{2\Omega\Delta} \sum_j \frac{c_j N_A \sigma_j z_j}{1 + \Gamma\sigma_j}. \tag{1.23}$$

The screening length Γ is defined by the implicit function Eq 1.24:

$$\Gamma^2 = \frac{e^2}{4k_B T \varepsilon} \sum_j c_j N_A \left(\frac{z_j - \zeta\sigma_j^2}{1 + \Gamma\sigma_j} \right)^2. \tag{1.24}$$

and

$$\Omega = 1 + \frac{\pi}{2\Delta} \sum_j \frac{c_j N_A \sigma_j^3}{1 + \Gamma\sigma_j}. \tag{1.25}$$

The only (fitting) parameters necessary in the MSA are the ionic radii, given in Table 1.1.

species	ionic diameter (Å)
Na^+	2.59
H^+	3.82
SO_4^{2-}	3.00
HSO_4^-	4.94
H_2SO_4	4.84
CO_3^{2-}	4.50
HCO_3^-	4.50
H_2CO_3	4.84
OH^-	3.99
Thb^{2-}	12.00
$HThb^-$	12.00
H_2Thb	12.00

Table 1.1: Diameter σ of all species used for the parameterization of the MSA. Thb denotes the dianionic form of thymol blue.

Equilibrium reactions Equilibrium reactions are considered for the acids (including water) via their equilibrium constants K. For the reaction:

$$n\mathrm{H}^+_{(aq)} + \mathrm{A}^{z}_{(aq)} \rightleftharpoons \mathrm{H_n A}^{z+n}_{(aq)}, \tag{1.26}$$

where A denotes an anion with (negative-valued) valence z, "aq" denotes a dissolved species, and n is the stoichiometric coefficient of H^+, the equilibrium is defined by:

$$K = \frac{a_{\mathrm{H_nA}}}{a_H^n a_A}. \tag{1.27}$$

For brevity, the valences of all species and the subscript "aq" have been omitted in the notation of Eq. 1.27. Table 1.2 shows the equilibrium constants at infinite dilution used in this work:

Acid	pK_a
H_2O	14.00
H_2SO_4	-3.00
HSO_4^-	1.90
H_2CO_3	6.52
HCO_3^-	10.40
H_2Thb	1.70
$HThb^-$	8.90

Table 1.2: pK_a values for the dissociation of the respective acid. Thb denotes the dianionic form of thymol blue.

1.3 Transport Properties and Their Prediction

Based on continuum mechanics and the idea of an ionic atmosphere imminent to the Debye-Hückel theory (and the MSA), Onsager and Fuoss developed models for the forces acting on ions during their transport [8]. The derivation and the respective models applied to diffusion coefficients, conductivities and the viscosity are still state of the art. Here, models based on the MSA were used. In the following, important models are briefly explained.

Ionic conductivity At infinite dilution, the velocity of an ion is proportional to an (external) electric field acting on it, where the proportionality is given by the mobility. At finite concentrations, the ion is additionally affected by other ions in its ionic atmosphere and the drag due to motion of the solvent (electrophoretic force). The ionic atmosphere of an ion (which is on average oppositely charged to it) moves in the opposite direction of the ion due to its charge. Thus, the centre of charge of the ion and its ionic atmosphere are separated, leading to a relaxation force which

opposes the (external) electric field partially and reduces its velocity.

Electrophoresis (superscript eph) and relaxation (superscript rel) reduce the effective conductivity of the ions in solution. The ionic equivalent conductivity of a ion as a function of both forces can be expressed by Eq. 1.28:

$$\lambda_k = \lambda_k^0 \left(1 + \delta_k^{eph}\right)\left(1 + \delta_k^{rel}\right) \tag{1.28}$$

The equations for δ^{eph} and δ^{rel} were developed by Roger *et al.* [6]. Here, the final equations to calculate the conductivity in multi-ion electrolytes based on single ion properties at infinite dilution are given for the sake of completeness. The electrophoretic effect δ^{eph} is given by Eq. 1.29:

$$\delta_k^{eph} = -\frac{k_B T}{3\pi z_k D_k^0 \eta^0}\left(\Xi_k + \frac{\pi N_A}{4}\sum_j c_j z_j \sigma_j^2 - \frac{\pi N_A}{6}\sum_j c_j \sigma_j^3 \Xi_k\right), \tag{1.29}$$

where

$$\Xi_k = \frac{\Gamma z_k}{1 + \Gamma \sigma_k} + \frac{\zeta \sigma_k}{1 + \Gamma \sigma_k}. \tag{1.30}$$

The relaxation forces of one ion depend on all other ions in solution. δ^{rel} is given by Eq. 1.32:

$$\delta_k^{rel} = -\frac{\kappa^2 e_k}{3}\sum_{p=1}^{s} \chi_k^p \sum_{j=1}^{s}\sum_{i=1}^{s} \frac{t_j \chi_j^p I_i (e_i u_i' - e_j u_j')}{e_i e_j (u_i' + u_j')} \frac{\sinh(\kappa\sqrt{q_p}\sigma_{ij})}{\kappa\sqrt{q_p}\sigma_{ij})}\Theta. \tag{1.31}$$

κ is the inverse Debye screening length with:

$$\kappa = \sqrt{\sum_j \frac{N_A c_j e_j^2}{\epsilon k_B T}}. \tag{1.32}$$

σ_{ij} are the distance of closest approach between the ions i and j, Eq. 1.33:

$$\sigma_{ij} = \frac{\sigma_i + \sigma_j}{2}. \tag{1.33}$$

I_k denotes the relative ionic strength of k, Eq. 1.34:

$$I_k = \frac{c_k e_k^2}{\sum_j^s c_j e_j^2}. \tag{1.34}$$

$\bar{u}'$ is the mean mobility defined by Eq. 1.35:

$$\bar{u}' = \sum_{j}^{s} I_j u'_j, \tag{1.35}$$

where the prime denotes that the mobility is divided by Avogadro's constant: $u'_k = u_k/N_A$. Using the notation above, the transport numbers t_k at infinite dilution can be expressed by Eq. 1.36:

$$t_k = \frac{I_k u'_k}{\bar{u}'}. \tag{1.36}$$

χ_k^p and N_p are defined by Eq. 1.37:

$$\chi_k^p = \sum_{j=1}^{s} \frac{N_p u'_k}{{u'_k}^2 - \alpha_p^2}, \tag{1.37}$$

and Eq. 1.38:

$$\frac{1}{N_p^2} = \sum_{j=1}^{s} \frac{t_j {u'_j}^2}{({u'_j}^2 - \alpha^2)^2}, \tag{1.38}$$

where the α_p are the roots of Eq. 1.39:

$$-2\bar{u}'\alpha \sum_{j=1}^{s} \frac{t_j}{{u'_j}^2 - \alpha^2} = 0. \tag{1.39}$$

To find the roots efficiently, Onsager showed that they follow Eq. 1.40:

$$0 = \alpha_1^2 < {u'_1}^2 < \alpha_1^2 < ... < \alpha_s^2 < {u'_s}^2 \tag{1.40}$$

Θ is given by Eq. 1.41:

$$\Theta = \int_{\sigma_{ij}}^{\infty} r \exp(\kappa\sqrt{q_p}\sigma_{ij}) h_{ij}^0 dr \tag{1.41}$$

$$\approx -\frac{e_i e_j \kappa\sqrt{q_p}\sigma_{ij} \exp(\kappa\sqrt{q_p}\sigma_{ij})}{4\pi\epsilon k_B T \left(\kappa^2 q_p + 2\Gamma\kappa\sqrt{q_p} + 2\Gamma^2 - 2\Gamma^2 Y\right)}. \tag{1.42}$$

and

$$Y = \left(\frac{\sum_j^s c_j N_A \frac{z_j^2}{(1+\Gamma\sigma_j)^2} \exp(\kappa\sqrt{q_p}\sigma_j)}{\sum_j^s \frac{c_j N_A z_j^2}{(1+\Gamma\sigma_j)^2}} \right). \tag{1.43}$$

Viscosity The increase of the relative viscosity (denoting the ratio of the electrolyte viscosity and the viscosity of the pure solvent) of electrolytes with concentration can be attributed to ionic long-range effects (LR), species-specific effects (s) and species-species interactions (s-s) [9]:

$$\eta_r = 1 + \eta_r^{\mathrm{LR}} + \eta_r^{\mathrm{s}} + \xcancel{\eta_r^{\mathrm{s-s}}} \tag{1.44}$$

η_r^{s-s}, showing a proportionality to I^2 or c^2, respectively, is neglected in this work. According to Onsager [8], the contribution of electrostatic (long-range) interactions of ions to the viscosity of the electrolyte is given by Eq. 1.45:

$$\eta_r^{\mathrm{LR}} = \frac{1}{\eta_0}\frac{Fe^2 10^8}{480\pi}\left(\frac{2N_A I}{\epsilon k_B T}\right)^{1/2}\left[\left(\sum_j \frac{I_j z_j}{\lambda_j}\right) - 4\mathbf{r}\xcancel{\sum_{n=0}^{\infty} c_n \mathbf{s}^n}\right], \tag{1.45}$$

In this work, the second term (giving the second order correction due to changing equivalent conductances) in the brackets of Eq. 1.45 is neglected. The second contribution to the relative viscosity η_r^s is given by Eq. 1.46:

$$\eta_r^{\mathrm{s}} = \sum_j c_j B_j, \tag{1.46}$$

where the B_j are ion specific parameters [9].

Diffusion coefficients The diffusion of ions can be discussed in terms of viscous, electrophoretic, and relaxation effects [10]. The electrolyte viscosity is assumed to affect the observed diffusion inversely, Eq. 1.47 [10]:

$$D_k \propto \frac{1}{\eta_r}. \tag{1.47}$$

The electrophoretic effect on diffusion coefficients was developed by Onsager and Fuoss, showing a correction by infinitely many terms for the diffusion coefficient, Eq. 1.48:

$$D = D^0 + \sum_{n=1}^{\infty} \Delta_n \tag{1.48}$$

However, depending on the considered ion valences, the sequence of Δ_n as well as the respective series ($\sum_{n=1}^{\infty} \Delta_n$) do not necessarily converge. Hence, Onsager and Fuoss suggested to only use the first two terms. Overall, the electrophoretic theory for diffusion coefficients in electrolytes is not satisfactory, especially for electrolytes including multivalent ions [10, p. 332]. Therefore, electrophoretic corrections are neglected for diffusion coefficients.

Relaxation effects were discussed by Chhih *et al.* [5] for the case of self-diffusion. However, in this work, relaxation effects are also neglected. After all, electrostatic interactions are considered *via* diffusion potentials. Hence, the effective diffusion coefficient considered in this work is given by Eq. 1.49

$$D_k = D_k^0 \frac{1}{\eta_r}. \tag{1.49}$$

2. Additional Experimental and Simulative Results

2.1 Experimental Evaluation

Figure 2.1 shows an exemplary photo of the electrolyte including the electrode reactions, the optically non-observable area, and the frame used for the evaluation.

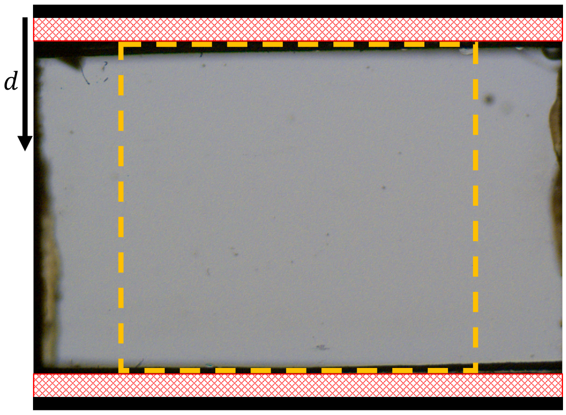

Figure 2.1: Exemplary photo of the electrolyte including the electrode reactions, the optically non-observable area (red shaded), and the frame used for the evaluation. At the cathode (top) pure oxygen is reduced, at the anode (bottom) hydrogen is oxidized.

Figure 2.2 shows the initial photo ($t = 0$ s) analysed for the evaluation of the location of the transition zone. The transition zone is detected wrongfully. This error might be due to shades close to the reactor parts.

Figure 2.3 shows the electrolyte including the evaluated transition zone from 0 to 220 s every 10 s.

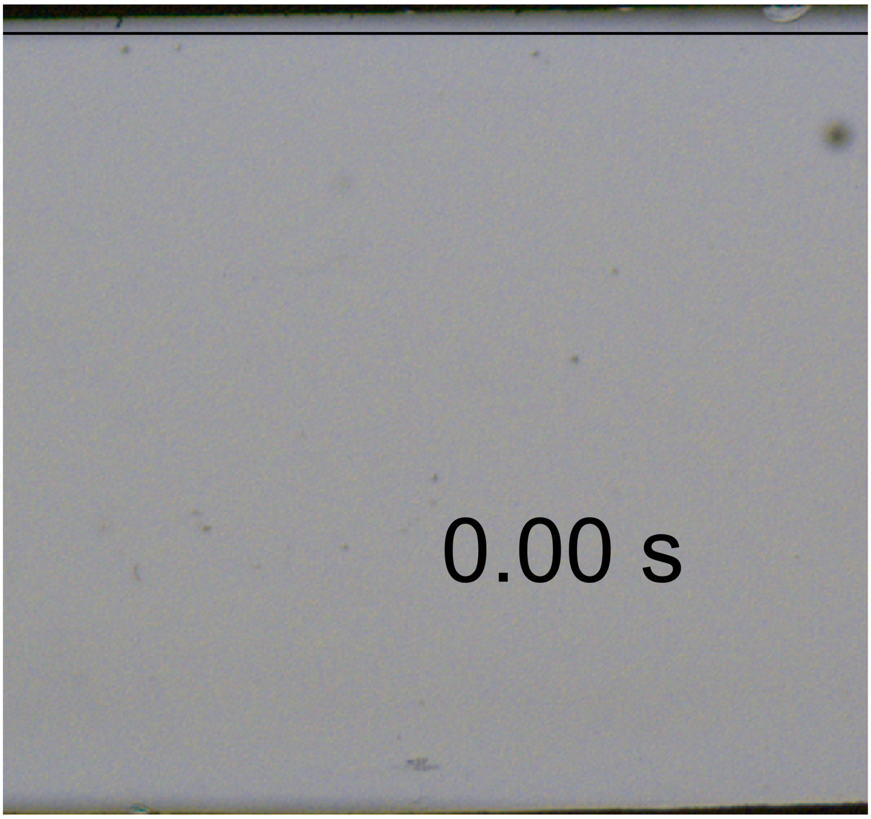

Figure 2.2: Experimental electrolyte image taken at 0 s and (wrongfully) detected transition zone. For the detection of the transition zone, the intensity of the blue component of the picture was compared to the red and green components. The erroneous detection of the transition zone might be due to the shade close to the reactor.

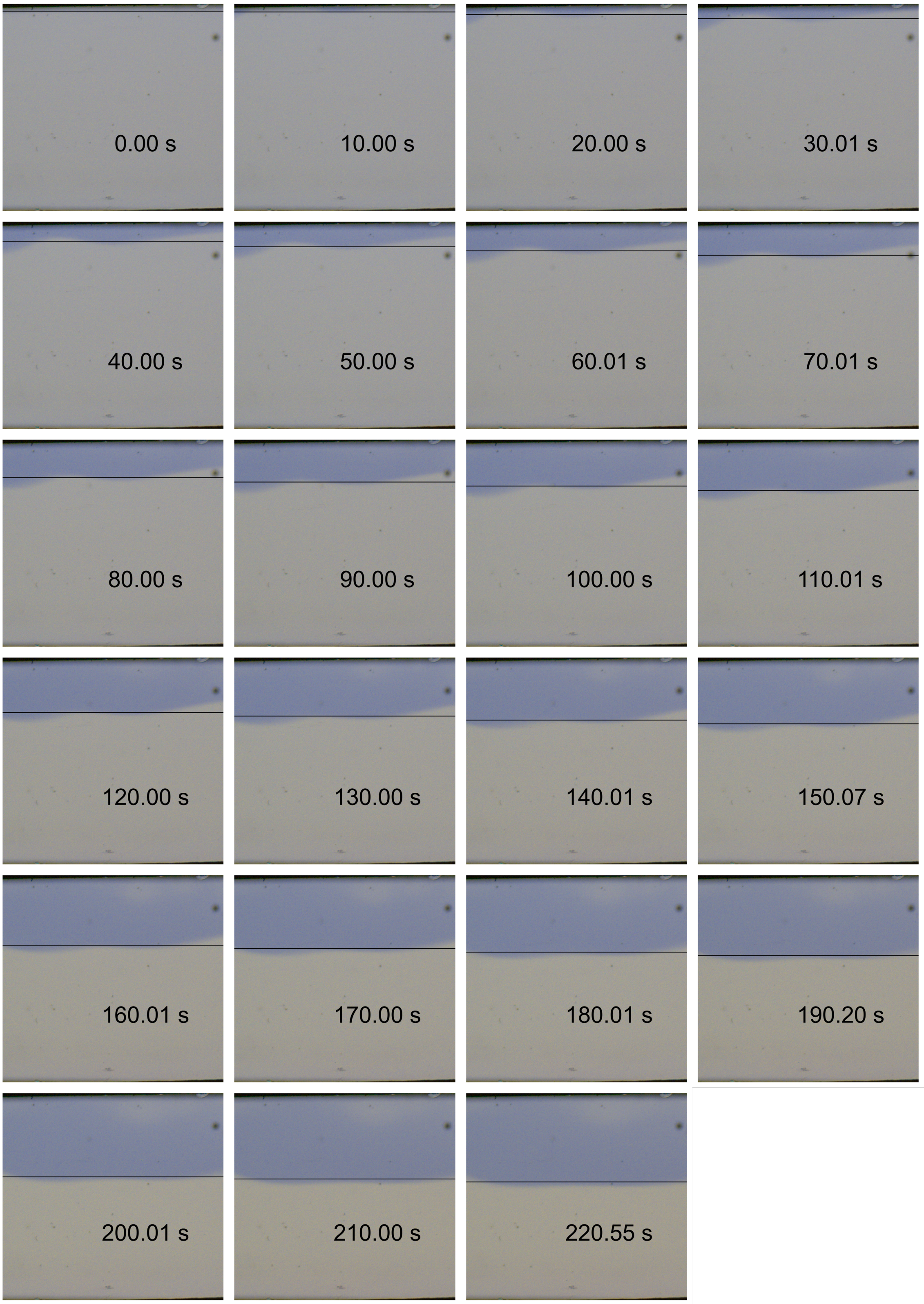

Figure 2.3: Experimental electrolyte images taken every 10 s including the respective detected location of the transition zone.

2.2 Simulative Results

Figure 2.4 shows the concentrations for S1 to S4 at A) 20 s, B) 50 s, C) 100 s, D) 200 s.

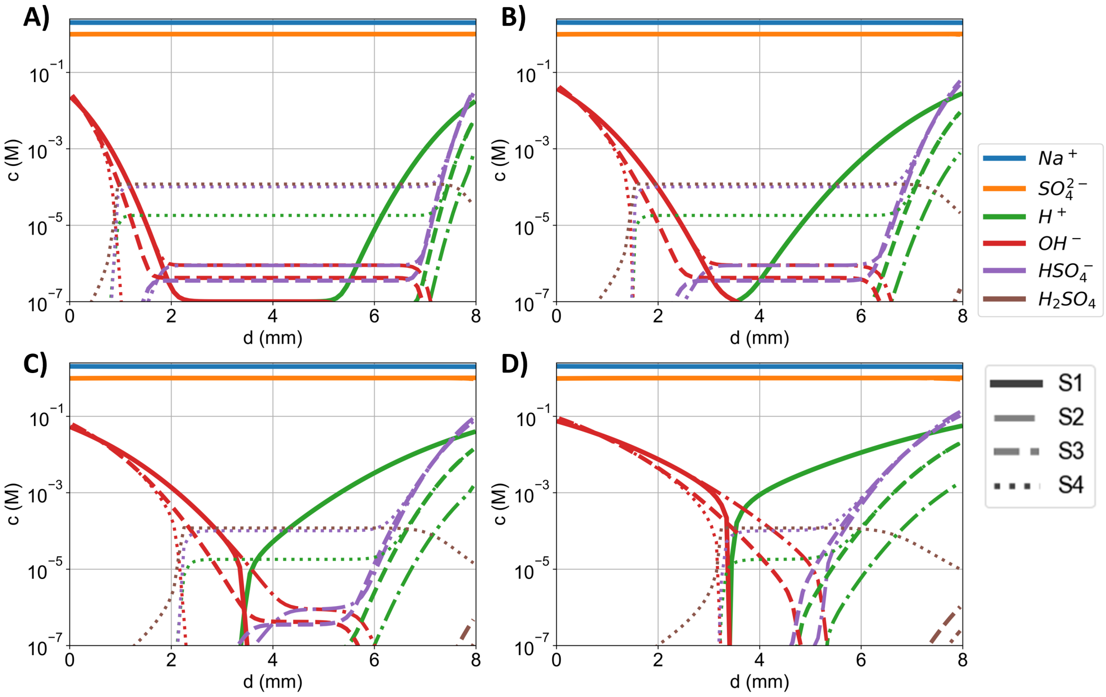

Figure 2.4: Simulation results for A) 20 s, B) 50 s, C) 100 s, D) 200 s.

Figure 2.5 shows the evaluated location of the transition zone including the conservative experimental uncertainty and the simulation results. Additionally, an exemplary linear interpolation for the transition zone between $t = 0$ s at $d = 0$ mm and $t = 20$ s at the computationally evaluated height is shown. The time increment for the simulations in Figure 2.5 was 0.01 s instead of 1 ms in the manuscript. As the results are basically identical, the time increment of 1 ms is justified.

Figure 2.6 shows the parameter variation of the concentration (0.01, 0.1, 1 M) and the current density (0.1, 1, 10 mA/cm^2 pH and as a function of time with a square-root scaled time axis. The scaled axis serves to illustrate the approximate square-root behaviour of pure one-dimensional diffusion and deviations from it at high current densities and low concentrations.

Figure 2.7 shows the parameter variation of the diffusion coefficient D^0 (values at infinite dilution divided by 10, times 1, times 10) and the current density (0.1, 1, 10 mA/cm^2 at 1 M, depicting a diffusion-limited propagation of the pH fronts.

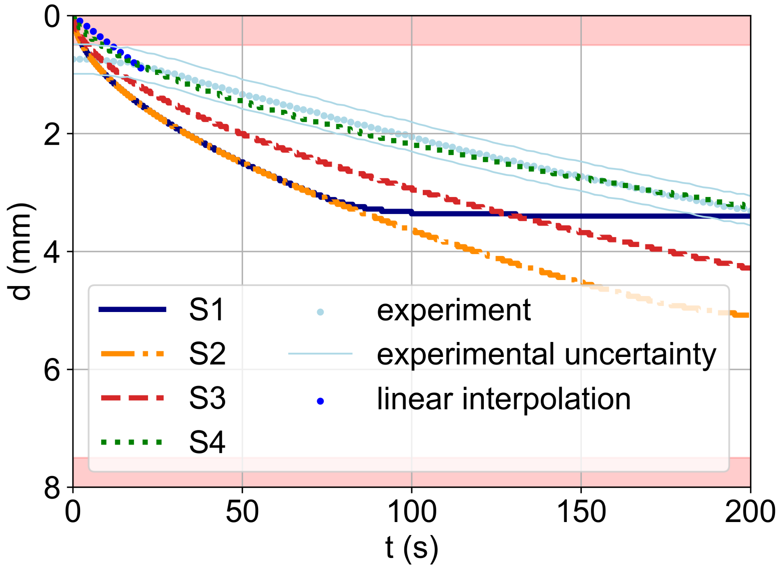

Figure 2.5: Evaluated location of the transition zone from simulation S4, and optical data including the uncertainty. The computationally evaluated location does not start at $d = 0$ as discussed in Figure 2.1. For a more useful comparison with the simulation, the blue dots show a linear interpolation between 0 and the optically evaluated data at 20 s. The red area depicts the non-observable region of the reactor. The time increment for the simulations was 0.01 s instead of the usual 1 ms in the manuscript.

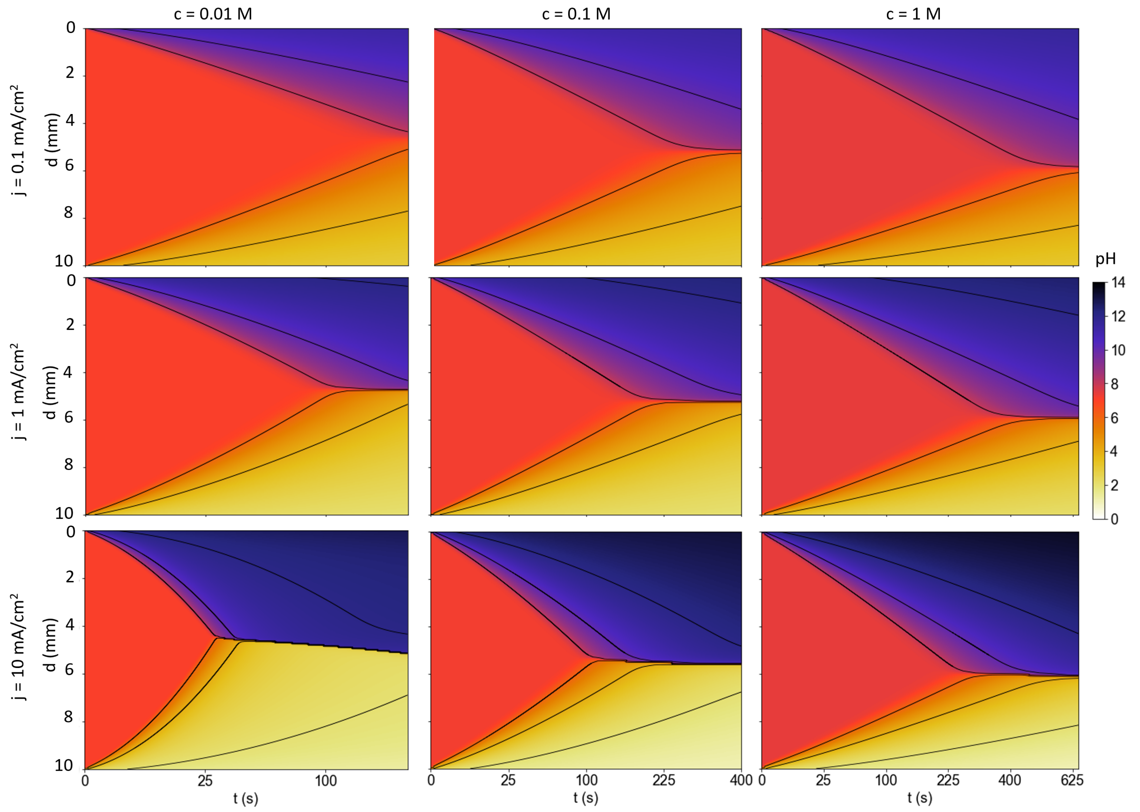

Figure 2.6: Simulation results for a parameter variation of the concentration (0.01, 0.1, 1 M) and the current density (0.1, 1, 10 mA/cm^2 pH and as a function of time with a square-root scaled time axis (compare to Fig. 8 in main publication).

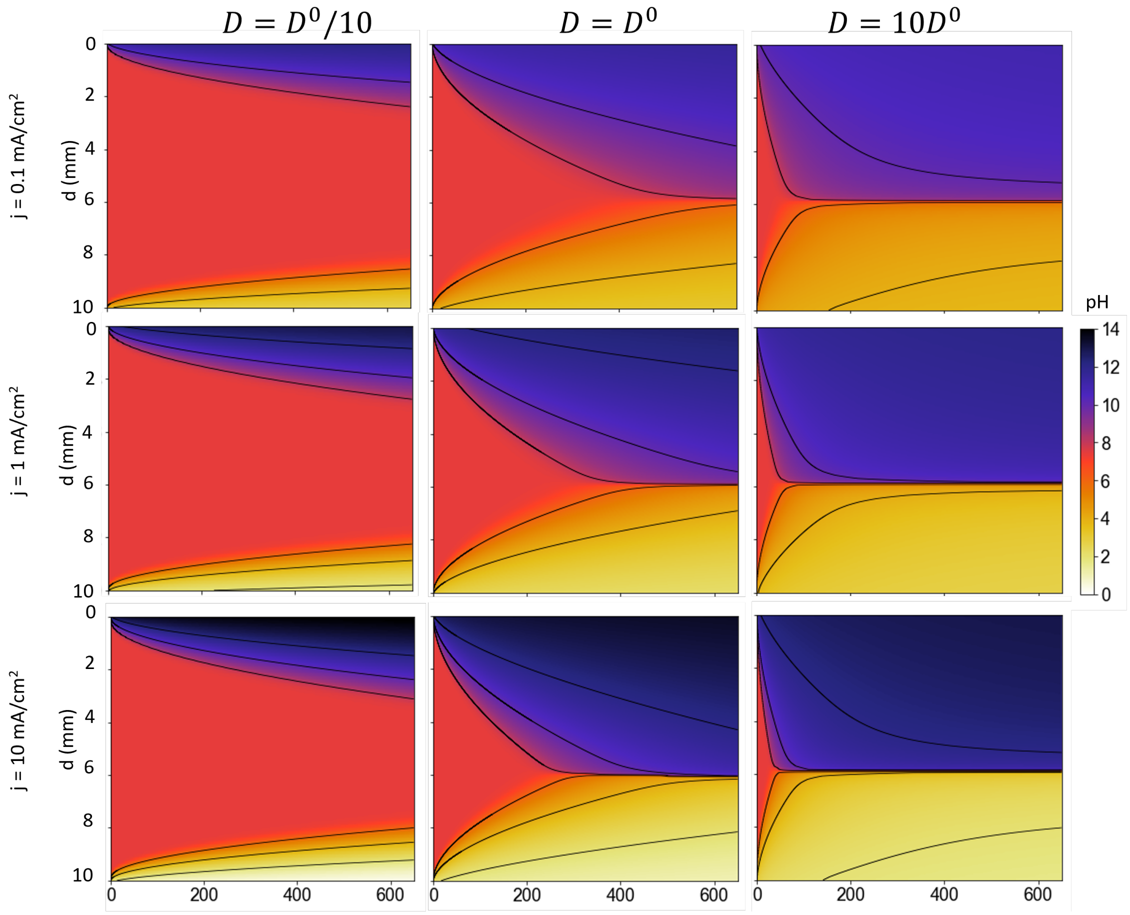

Figure 2.7: Simulation results for a parameter variation of the diffusion coefficient D^0 (values at infinite dilution divided by 10, times 1, times 10) and the current density (0.1, 1, 10 mA/cm^2 at a Na_2SO_4 concentration of 1 M.

2.3 Effect of dissolved CO_2

The effect of dissolved CO_2 on the ion transport and pH profiles was investigated. The pK_a values for the equilibria between dissolved H_2CO_3, HCO_3^-, and CO_3^{2-} are given in Table 1.2. Figure 2.8 shows a comparison of S4 (simulations based on the MSA and considering the concentration of thymol blue) with simulations additionally including 0.05 mM dissolved H_2CO_3. The differences regarding the pH (Figure 2.9) as well as for the concentrations over time are negligible. The dissolved H_2CO_3 may be introduced through contact of the electrolyte with air. Figures 2.10 and 2.11 show a comparison of S3 (simulations based on the MSA, not considering thymol blue) with simulations additionally including 0.05 mM dissolved H_2CO_3 for the concentrations and the pH, respectively. The differences regarding the pH (Figure 2.9) are comparable to the difference between S3 and S4 (thymol blue instead of H_2CO_3). This similarity is due to the rather similar pK_a values for the dissociation equilibria of H_2CO_3 and thymol blue, and similar used concentrations. Hence, the impact of dissolved CO_2 from the air is assumed to be negligible in this study.

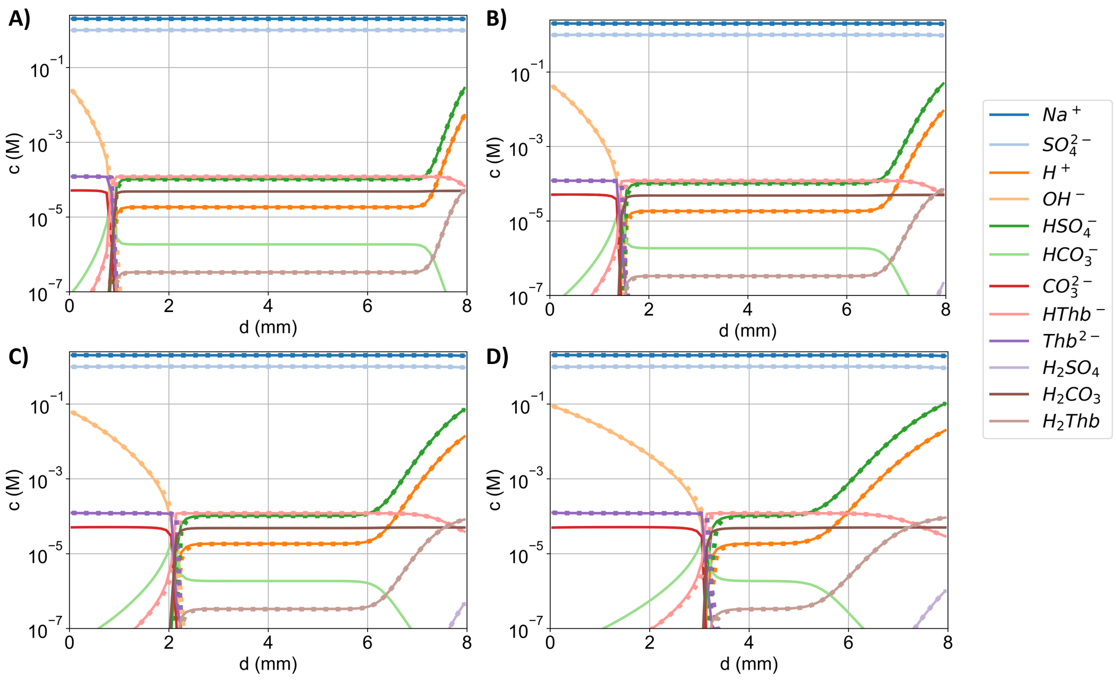

Figure 2.8: Comparison between results of S4 (dotted) and S4 including 0.05 mM dissolved H_2CO_3 (solid) for A) 20 s, B) 50 s, C) 100 s, D) 200 s. The concentrations of all common species in both cases are almost similar.

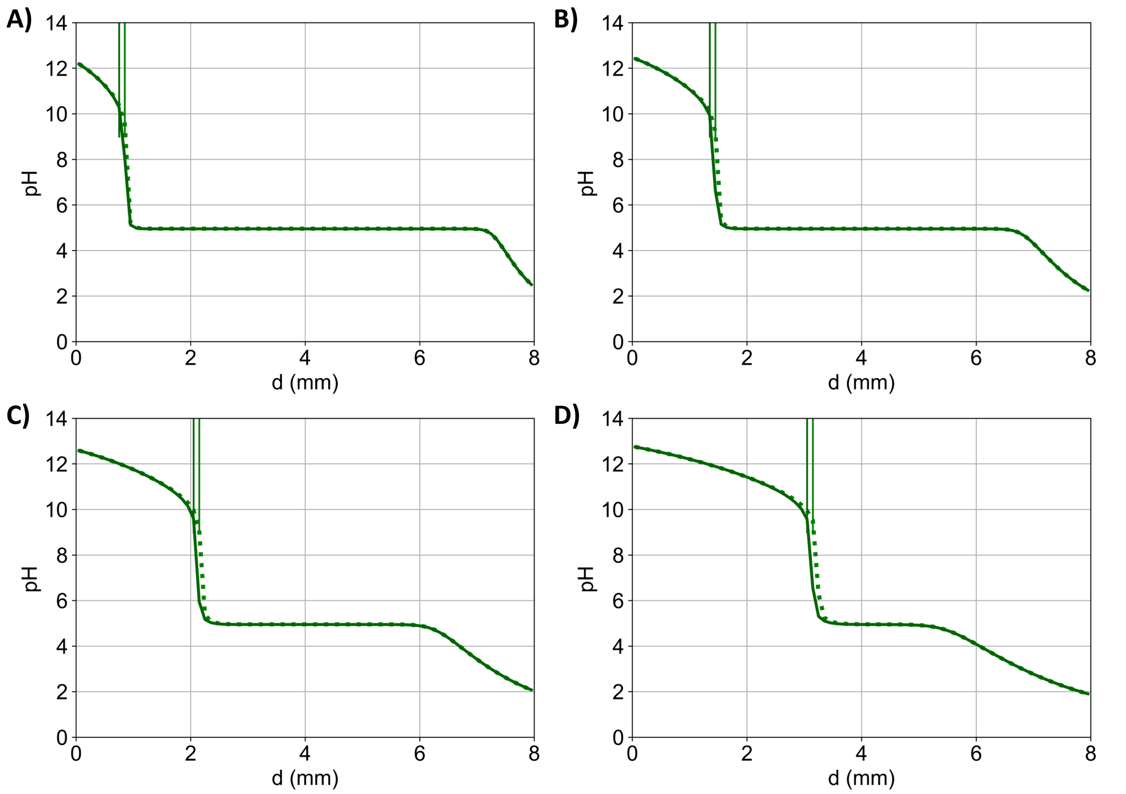

Figure 2.9: Comparison between results of S4 (dotted) and S4 including 0.05 mM dissolved H_2CO_3 (solid) for A) 20 s, B) 50 s, C) 100 s, D) 200 s. The pH profiles in both cases are almost similar.

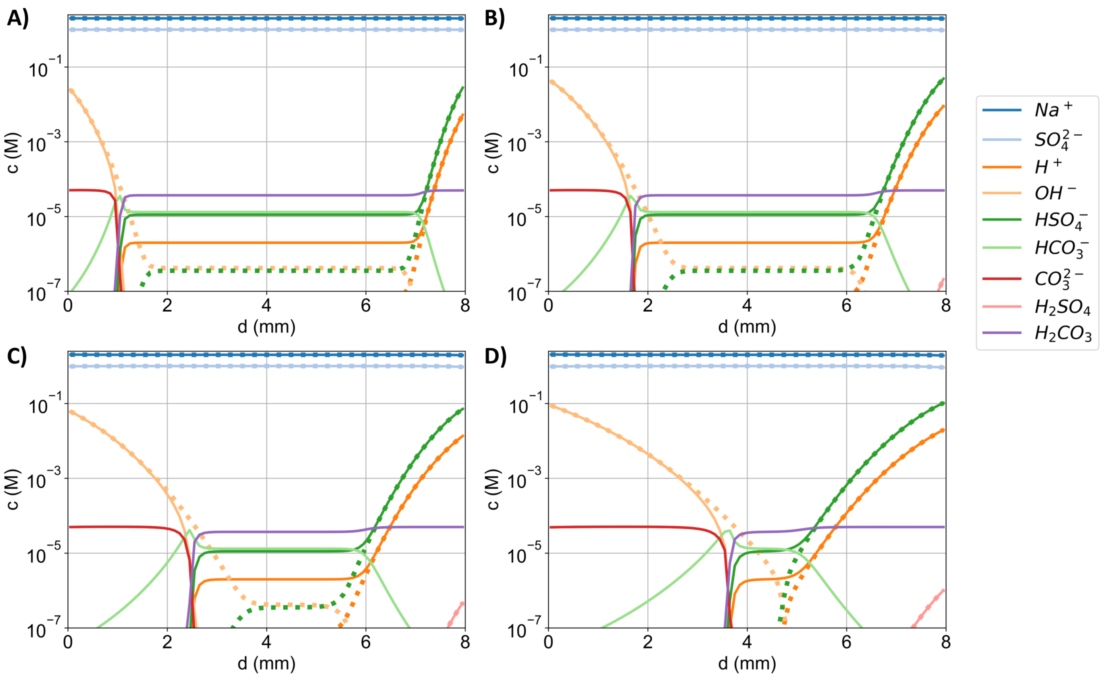

Figure 2.10: Comparison between results of S3 (dotted) and S3 including 0.05 mM dissolved H_2CO_3 (solid) for A) 20 s, B) 50 s, C) 100 s, D) 200 s. The concentrations of all common species in both cases are almost similar.

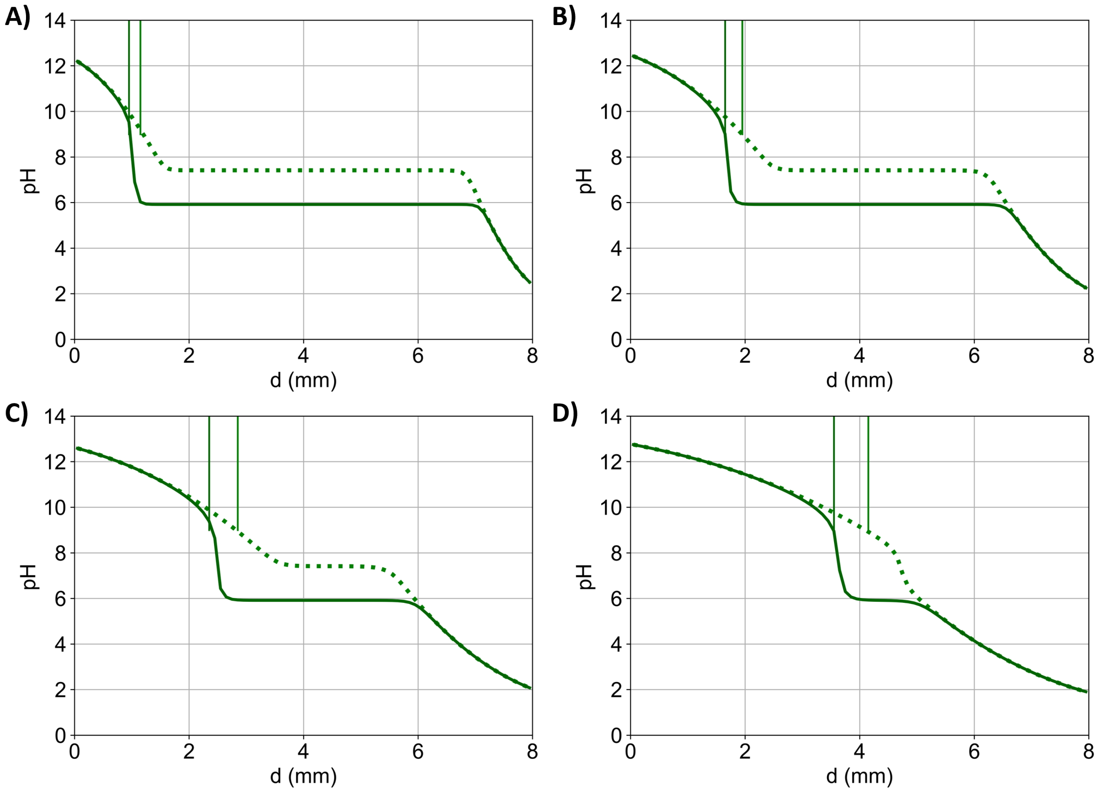

Figure 2.11: Comparison between results of S3 (dotted) and S3 including 0.05 mM dissolved H_2CO_3 (solid) for A) 20 s, B) 50 s, C) 100 s, D) 200 s. The pH profiles differ significantly in the near-neutral area.

Bibliography

(1) Nernst, W. *Zeitschrift für physikalische Chemie* **1888**, *2U*, 613–637.

(2) Planck, M. *Annalen der Physik* **1890**, *276*, 561–576.

(3) Planck, M. *Annalen der Physik* **1890**, *275*, 161–186.

(4) Blum, L. *Molecular Physics* **1975**, *30*, 1529–1535.

(5) Chhih, A.; Turq, P.; Bernard, O.; Barthel, J. M. G.; Blum, L. *Berichte der Bunsengesellschaft für physikalische Chemie* **1994**, *98*, 1516–1525.

(6) Roger, G. M.; Durand-Vidal, S.; Bernard, O.; Turq, P. *The journal of physical chemistry. B* **2009**, *113*, 8670–8674.

(7) Carnahan, N. F.; Starling, K. E. *The Journal of Chemical Physics* **1969**, *51*, 635–636.

(8) Onsager, L.; Fuoss, R. M. *The Journal of Physical Chemistry* **1932**, *36*, 2689–2778.

(9) Lencka, M. M.; Anderko, A.; Sanders, S. J.; Young, R. D. *International Journal of Thermophysics* **1998**, *19*, 367–378.

(10) Robinson, R. A.; Stokes, R. H., *Electrolyte solutions*, 2. rev. ed.; Dover books on chemistry; Dover Publications: Mineola, NY, 2002.
